# Stratified Care for Musculoskeletal Conditions in Primary Care: A Prospective Sequential Cohort Study of the STarT MSK Tool and the eSELF-MSK Virtual Self-Management Program

**DOI:** 10.3390/jcm15208105

**Published:** 2026-10-19

**Authors:** Norah AlMuhana, Dalia Alimam, Walaa Moslem, Ibraheem Alsmari, Fahad Alfarraj, Ameerah S. Alharthi, Muteb Aldosari, Abdullah Alassaf, Hamad M. Aloraini, Hana Alsobayel

**Affiliations:** 1 Department of Physiotherapy, Prince Sultan Military Medical City (PSMMC), Ministry of Defense Health Services, Riyadh 11159, Saudi Arabia; walaa.moslem@gmail.com (W.M.); ffalfarraj@gmail.com (F.A.); ameermine2@gmail.com (A.S.A.); 2 Department of Rehabilitation Health Sciences, College of Applied Medical Sciences, King Saud University, Riyadh 11451, Saudi Arabia; dalimam@ksu.edu.sa (D.A.); ptmaldosari@outlook.com (M.A.); hsobayel@ksu.edu.sa (H.A.); 3 Department of Family and Community Medicine, Prince Sultan Military Medical City (PSMMC), Ministry of Defense Health Services, Riyadh 11159, Saudi Arabia; dr.ibraheem@gmail.com (I.A.); alassaf.an@gmail.com (A.A.); hamad.aloraini8@gmail.com (H.M.A.)

**Keywords:** musculoskeletal disorders, stratified care, primary care, telerehabilitation, self-management, Saudi Arabia, STarT MSK Tool

## Abstract

**Background/Objectives:** To compare changes in pain intensity, musculoskeletal health and function, and psychosocial risk between usual care and implementation of a bundled STarT MSK-guided care pathway among low-risk adults. **Methods:** In this non-randomized sequential cohort study, the usual-care cohort was recruited before clinician training and pathway implementation, followed by recruitment of the stratified-care cohort. Both cohorts comprised adults classified as low risk by the STarT MSK Tool. The pathway combined risk classification feedback, clinician training, one in-person physiotherapy assessment, education and exercise prescription, supported self-management, and five weekly virtual consultations. NPRS and MSK-HQ, the prespecified primary outcomes, and ÖMPQ, a prespecified secondary longitudinal outcome, were assessed at baseline, 6 weeks, and 12 weeks and analyzed using linear mixed-effects models; satisfaction was a secondary outcome assessed at 12 weeks. **Results:** Of 750 patients screened, 136 were enrolled: 63 in usual care and 73 in stratified care. At 12 weeks, the bundled-pathway cohort showed greater improvement than usual care in NPRS (−2.12; 95% CI, −2.95 to −1.29), MSK-HQ (+7.08; 95% CI, +3.70 to +10.47), and ÖMPQ (−27.83; 95% CI, −37.90 to −17.76). Clinically important pain reduction occurred in 68.3% versus 25.9% of participants, and 88.9% versus 51.7% reported satisfaction with care. **Conclusions:** Implementation of the bundled STarT MSK-guided eSELF-MSK pathway was associated with greater 12-week improvements than usual care among low-risk patients. Because the pathway combined multiple components, their independent contributions cannot be separated. The non-randomized design, unblinded participants, self-reported outcomes, and possible residual confounding preclude causal conclusions and support further randomized evaluation.

## 1. Introduction

Musculoskeletal (MSK) disorders are the leading cause of disability worldwide and one of the most common reasons for primary care consultation in high- and lower-income settings alike [1]. In Saudi Arabia, the burden is acute: drawing on Global Burden of Disease data, the age-standardized prevalence of MSK disorders reached 19.05% (95% UI 17.96–20.22) by 2019, with MSK conditions ranking second among all causes of disability in the Gulf Cooperation Council region [2]. Saudi Arabia recorded one of the largest increases in MSK-related disability rates in the Middle East and North Africa (MENA) and Brazil, Russia, India, China, South Africa, and other major emerging economies (BRICS+) countries between 1990 and 2021 [3,4]. Potential contributors include population aging, obesity, and changes in activity patterns. A recent national Saudi report found that MSK disorders accounted for 37% of referrals to physical therapy [5].

Despite this burden, MSK management in Saudi primary care remains reactive and largely uninformed by prognostic risk. A cross-sectional survey of 283 primary care physicians across Riyadh’s three healthcare sectors found that 37.8% rarely referred MSK patients to physiotherapy, more than half were unfamiliar with any prognostic stratification tool, and radiology was the dominant management response regardless of clinical complexity [6]. These findings reflect a structural gap between available evidence and clinical practice. Guidelines and overviews consistently recommend education, exercise, and psychosocial interventions as core MSK treatments, with imaging and invasive options used selectively [7,8,9]. Consistent with this broader evidence-practice gap, a related pre-implementation study conducted within the present research program found low concordance between physician management decisions and STarT MSK risk-matched recommendations, including among patients classified as low risk [10]. Because that analysis examined clinical decision-making rather than patient outcomes, it provides contextual implementation evidence but not evidence on the clinical outcomes of the eSELF-MSK pathway.

Stratified care offers a systematic response to this gap. Stratified care classifies patients by their prognostic risk of persistent pain and disability, then matches treatment intensity accordingly. This directs physiotherapy and specialist resources to the patients most likely to benefit [11,12]. The Keele STarT MSK Tool—a validated 10-item self-report instrument covering the five most common MSK presentations (back, neck, shoulder, knee, and multisite pain)—classifies patients into low, medium, and high risk groups and links each to risk-matched treatment recommendations [13,14]. A psychometrically validated Arabic translation of the tool is available and was used in the present study [15]. Construct and predictive validity of the Keele STarT MSK Tool have also been demonstrated outside the United Kingdom, in a United States outpatient physical therapy cohort, in which risk-stratified subgroups differed in pain intensity, pain interference, and health-related quality of life from intake through 6-month follow-up, supporting the tool’s validity beyond its original UK development and validation setting [16]. A definitive evaluation of this approach in a UK cluster-randomized trial across 24 general practices found that stratified care changed clinician behavior—increasing physiotherapy referrals, reducing prescriptions, and prompting greater attention to psychosocial factors—even when it did not produce statistically significant improvements in pain or function over usual care [17,18].

Two established precedents—the IMPaCT Back study in UK family practice [19] and the SPLIT program in Portuguese primary care [20]—showed that a prospective sequential cohort design can provide a real-world framework for evaluating implementation of STarT-family stratified care in routine clinical settings. Both studies enrolled patients across all risk strata (low, medium, and high) and showed meaningful changes in clinician behavior alongside modest improvements in disability, pain, or related outcomes: IMPaCT Back reported a small improvement in disability (Roland–Morris Disability Questionnaire) and reduced work absence, while SPLIT reported improvements in disability, pain, and quality of life. However, neither addressed Saudi Arabia, the Arab world, or the STarT MSK Tool’s broader five-site scope. Previous implementation research shows that successful adoption of evidence-based musculoskeletal care depends on health-system organization, workforce capacity, provider practice patterns, and patient empowerment. Ignoring these factors can limit how well primary care adopts guideline-based care [21].

Telerehabilitation, the remote delivery of rehabilitation assessment and treatment via telecommunications technologies, such as real-time videoconferencing, has been proposed to expand access to MSK care where in-person services are limited [22,23]. For low-risk patients, the STarT MSK stratified-care pathway recommends supported self-management rather than intensive physiotherapy management [12,17]. Hybrid telerehabilitation, which combines an initial in-person assessment with subsequent virtual follow-up, has shown comparable satisfaction and non-inferior clinical outcomes relative to fully in-person physiotherapy for MSK conditions [24,25]. A recent Australian feasibility and pilot randomized controlled trial also combined Keele STarT MSK-based stratification with telehealth delivery. Patients and clinicians found the stratification approach acceptable, and it was also associated with a shorter median wait time to the first appointment (16 days) than usual care (29 days). However, the study did not meet its recruitment and consent feasibility targets, and no intervention participant was classified as low risk [26]. Accordingly, the eSELF-MSK program was developed as a hybrid supported self-management intervention for patients classified as low risk. Its design was informed by a preceding qualitative study of Saudi adults with MSK pain, which identified preferences for an initial in-person assessment session, culturally relevant guidance on prayer-position modification, and accessible health information [27].

This study aimed to compare changes in pain intensity, MSK health and function, and psychosocial risk between usual care and implementation of a bundled STarT MSK-guided care pathway for adults classified as low risk. The implemented pathway included communicating the risk classification to clinicians, clinician training, an initial physiotherapy assessment, education, exercise prescription, supported self-management, and five virtual follow-up consultations. Patient satisfaction with care was evaluated as a prespecified secondary outcome. Because both cohorts were screened using the STarT MSK Tool and enrollment was restricted to low-risk participants, the study was not designed to isolate the tool’s independent contribution or the effect of risk classification.

## 2. Materials and Methods

### 2.1. Study Design

A prospective sequential cohort study comprising a usual-care cohort, followed by a training and implementation phase, and subsequently a stratified-care cohort.

### 2.2. Setting

The study was conducted at the Primary Healthcare (PHC) Center at Prince Sultan Military Medical City (PSMMC), Riyadh, Saudi Arabia. The PSMMC is a large tertiary healthcare institution serving the beneficiaries of the Ministry of Defense Health Services. The PHC center comprises 19 family medicine clinics operating daily from 08:00 to 20:00 and manages up to 400 patient visits each day, providing a high-volume primary care setting for MSK presentations. The research team worked in a dedicated physiotherapy clinic adjacent to the clinic triage and booking area throughout the study. The physiotherapy clinic served as the study site, where the team conducted the initial physiotherapy assessment and delivered treatment/self-treatment. The research team maintained clinical documentation using the RABET Electronic Health Record (EHR) system, which serves and links all Medical Services Directorate (MSD) health facilities at PSMMC and is powered by Cerner Corporation (North Kansas City, MO, USA) [28].

During the stratified-care cohort, follow-up consultations were delivered through the Telerehabilitation Center at the main PSMMC hospital using ANAT Platform, an online platform for health practitioners registered with the Saudi Commission for Health Specialties and approved by the Saudi Ministry of Health [29]. Sehhatek, a Ministry of Defense Health Services platform developed for PSMMC personnel and their families [30], automatically sent appointment notifications to participants, enabling them to join scheduled video consultations remotely. When participants missed a scheduled virtual appointment, staff contacted them by phone to facilitate adherence to the follow-up session.

### 2.3. Participants and Eligibility Criteria

Patients were eligible if they were aged 18 years or above, presented to primary care with an MSK complaint involving pain affecting the back, neck, shoulder, knee, or multiple sites, corresponding to the five presentations covered by the validated Keele STarT MSK Tool, and were classified as low risk using the Keele STarT MSK Tool (total score ≤ 4). Low-risk status was an inclusion criterion because stratified-care guidelines recommend supported self-management for low-risk patients, whereas medium- and high-risk patients should receive more intensive physiotherapy or specialist management, which the eSELF-MSK program was not designed to provide.

We excluded patients if they were unable to use a smartphone or tablet or complete questionnaires in Arabic; had a specific pathology, such as an inflammatory arthropathy, or a severe neurological deficit likely to require a different treatment approach; had undergone spinal surgery within the previous six months; had severe depression or another significant psychiatric condition, or were pregnant; or had severe comorbidity. No screening score or numerical threshold was used to apply these criteria. Research assistants assessed eligibility from information in the electronic medical record and the treating physician’s clinical assessment at the index consultation. Severe depression referred to a documented depressive or other significant psychiatric condition considered likely to impair informed participation, engagement with supported self-management, or completion of follow-up assessments. Severe comorbidity referred to a documented medical condition considered to make participation in the exercise and hybrid telerehabilitation program unsafe or impracticable, or to require a substantially different clinical pathway. These criteria were applied because they identify patients whose presentation, safety profile, or capacity to engage in a virtual self-management program would likely require a different treatment approach or assessment method than the eSELF-MSK pathway provides.

Research assistants identified eligible patients upon arrival and screened them at their first visit using a standardized form. Those meeting all criteria provided written informed consent before enrollment. All enrolled participants were in the low-risk category; therefore, the findings do not extend to medium- or high-risk patients.

### 2.4. Sample Size

An a priori sample-size calculation was conducted in G*Power version 3.1.9.4 (Heinrich Heine University Düsseldorf, Düsseldorf, Germany); a repeated-measures analysis of variance (ANOVA) with a within–between interaction was used as a planning approximation. The calculation assumed a correlation of 0.5 among repeated measures (further details in Supplementary Material S1). Based on previous stratified care studies, particularly Foster et al. [31] and Hill et al. [13], a small-to-moderate effect size (f = 0.15) was assumed, together with a two-sided significance level of 0.05, 80% statistical power, two study groups, and three assessment time points (baseline, 6 weeks, and 12 weeks) [13,31]. The minimum required sample size was 74 participants (37 per cohort). To account for an anticipated 20% attrition rate, we increased the recruitment target to 93 participants (approximately 47 per cohort). The study enrolled 136 participants: 63 during the usual-care cohort and 73 during the stratified-care cohort, exceeding the overall recruitment target.

### 2.5. Screening and Recruitment

**Figure 1:**
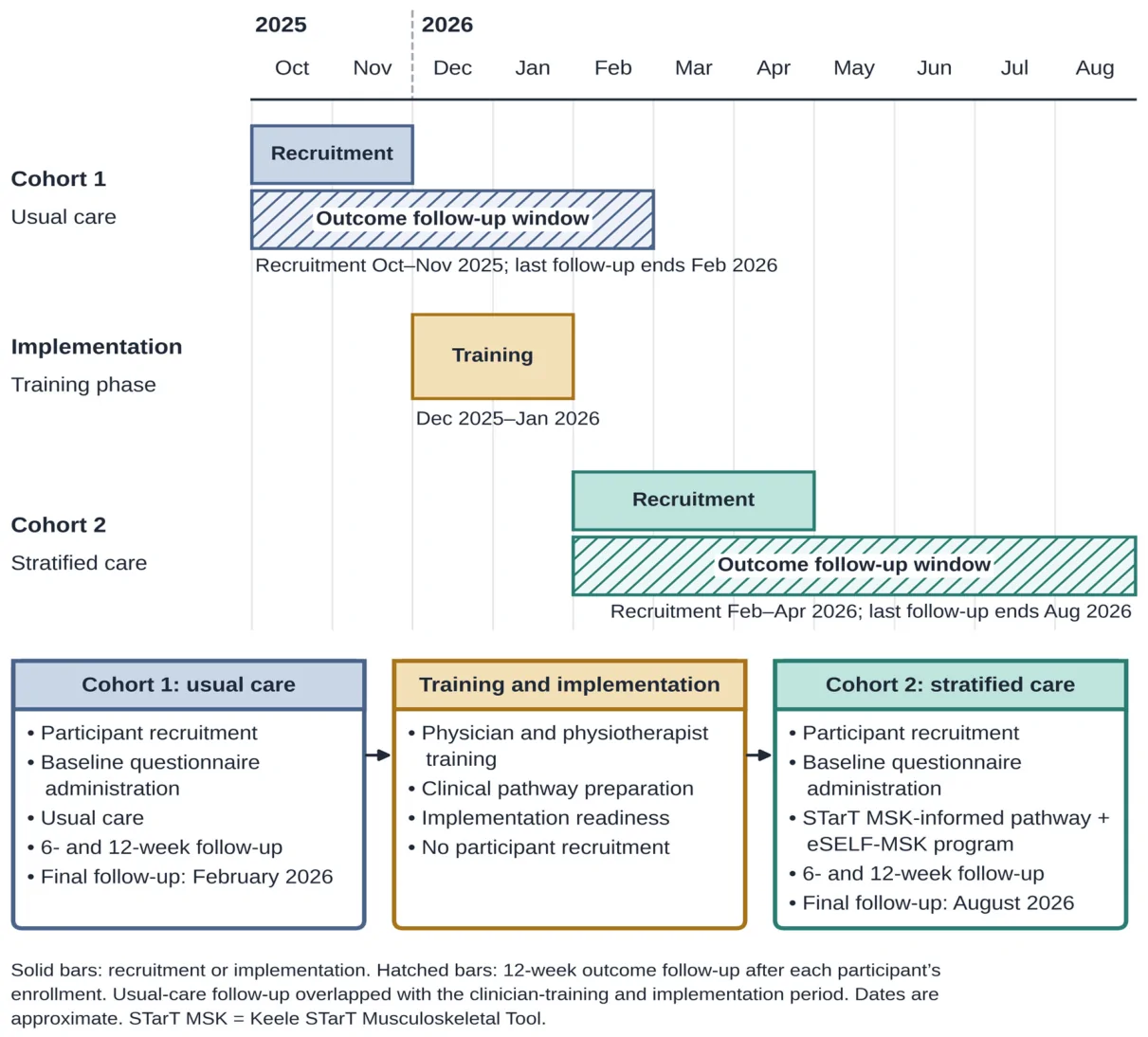
Recruitment, implementation, and follow-up timeline for the sequential cohort study. Solid bars indicate recruitment or implementation periods; hatched bars indicate the cohort-wide outcome follow-up windows through 12 weeks after each participant’s enrollment. Usual-care follow-up overlapped with the clinician-training and implementation period. Dates are approximate. Some stratified-care 12-week assessments were delayed by approximately one week because of the Eid al-Adha holiday.

Recruitment was undertaken in two sequential implementation cohorts. The usual-care cohort (Cohort 1) was recruited between October and November 2025. After recruitment, a two-month implementation period (December 2025 to January 2026) focused on training primary care physicians and physiotherapists and integrating the stratified care pathway into routine clinical practice. Physician training included an in-person orientation session led by the principal investigator, covering the biopsychosocial rationale for stratified care, the STarT MSK Tool’s structure and scoring, the color-coded referral pathway, and the eSELF-MSK program. Point-of-care materials, including a referral-guide brochure, were placed in consulting rooms, and the principal investigator visited participating clinics before each recruitment phase to reinforce the pathway. Recruitment for the stratified-care cohort (Cohort 2) began in February 2026, immediately after the implementation period ended, and continued through the end of April 2026. Recruitment paused during the holy month of Ramadan (the Islamic month of daytime fasting, associated with changes in diet, sleep, and clinic attendance) and the subsequent Eid al-Fitr holiday, then resumed and concluded in April 2026. Two participants completed their baseline questionnaires in early May 2026 after the initial in-person consultation but before their first virtual follow-up session. Participants in both cohorts were followed for 12 weeks after enrollment. Consequently, outcome follow-up continued until February 2026 for the usual-care cohort and August 2026 for the stratified-care cohort. Follow-up for the stratified-care cohort extended into August 2026, and the final 12-week assessments of some stratified-care participants were delayed by about one week because of the Eid al-Adha holiday. Although follow-up periods overlapped, participant recruitment and intervention delivery occurred sequentially, and participants enrolled in the usual-care cohort were not exposed to the stratified-care pathway or the eSELF-MSK program (Figure 1).

Recruitment for both cohorts was conducted in the primary care triage and appointment-booking area. Two trained research assistants (one male and one female), who received standardized training from the principal investigator in administering the Arabic Keele STarT MSK Tool [15] and study procedures, screened consecutive patients attending the primary care clinics. Eligible patients filled in the STarT MSK Tool by scanning a QR code linked to a purpose-built Google Form used only to determine participant eligibility and to automatically calculate the STarT MSK risk category. It collected only the participant’s medical record number and mobile telephone number to confirm eligibility and facilitate follow-up; it did not record participant names. After eligibility screening, the study entered all the secure REDCap (Research Electronic Data Capture) database (version 13.7.9; Vanderbilt University, Nashville, TN, USA), which served as the study’s primary data management system. Access to the Google Form responses was restricted to authorized members of the research team. The electronic form automatically calculated the STarT MSK score and immediately displayed the corresponding risk category. Participants without a smartphone or internet access completed the questionnaire using a tablet provided by the research team. Research assistants, rather than treating physicians, performed screening to ensure standardized prognostic classification of all potentially eligible participants and to minimize variability associated with physician workload, time constraints, and differing familiarity with the STarT MSK Tool. Patients classified as low risk who met the study eligibility criteria were referred to the physiotherapy clinic for eligibility confirmation, written informed consent, baseline assessment, and enrollment into the study. During the usual-care cohort, STarT MSK risk classifications were recorded solely for research purposes and were not disclosed to treating physicians. Consequently, physicians managed patients according to routine clinical practice without knowledge of patients’ prognostic risk classification. During the stratified-care cohort, the same screening procedures were maintained; however, STarT MSK risk classifications were communicated to treating physicians using a color-coded referral card (blue = low risk, yellow = medium risk, red = high risk). The card enabled physicians to identify each patient’s prognostic risk category at a glance and supported risk-informed clinical decision-making without interpreting the numerical STarT MSK score. The card was decision support only: physicians were not required to follow a particular pathway and retained their clinical judgment. Patients classified as low risk were referred to the physiotherapy clinic for enrollment in the eSELF-MSK program, whereas patients classified as medium or high risk continued to receive routine physician management according to the stratified referral pathway.

### 2.6. Implementation

During the usual-care phase, the research physiotherapist’s involvement was limited to confirming eligibility, obtaining informed consent, and administering the study questionnaires. The research physiotherapist did not provide clinical advice, education, exercise prescription, or treatment. The study recorded management decisions made by the primary-care physician during the index consultation using predefined, non-mutually exclusive categories, including medication prescription (type not recorded), MSK imaging, referral to conventional physiotherapy, referral to secondary care, a follow-up appointment, or another decision (advice and education were not a predefined field and were recorded as free text under “other decision”). When prescribed, conventional physiotherapy was delivered in a separate facility rather than at the participating primary care center. The study recorded management decisions made during the index consultation but did not systematically verify subsequent attendance, treatment content, number of conventional physiotherapy sessions, medication use after the index consultation, or additional healthcare consultations. All 73 participants in the stratified-care cohort received an official physiotherapy referral and were enrolled in the eSELF-MSK program. Participants recruited to the usual-care cohort remained in that cohort throughout follow-up and were not subsequently enrolled in the stratified-care pathway or the eSELF-MSK program. The research team maintained regular contact with participants during follow-up and identified no crossover between cohorts. Therefore, direct intervention contamination was unlikely, although we did not systematically capture healthcare consultations outside the study pathway.

Primary care physicians attended one structured orientation session covering STarT MSK principles, the color-coded referral pathway, and the eSELF-MSK program during the intervening training-and-implementation period between the usual-care and stratified-care cohorts. The team distributed a referral guide brochure to all physicians. Before each session for the stratified-care cohort, the principal investigator visited participating clinics to reinforce the referral pathway, answer physicians’ questions, and confirm the color-coded referral cards were clear. Physiotherapists completed structured training, including in-person educational sessions, practical demonstrations of condition-specific exercise modules, and mock virtual consultations with feedback; competency was verified before implementation began in the stratified-care cohort. All materials were accessible via the PSMMC E-Knowledge Portal throughout the stratified-care cohort’s implementation period. The corresponding author can provide complete training materials upon request.

### 2.7. The eSELF-MSK Program

The eSELF-MSK program is a six-week hybrid telerehabilitation intervention developed specifically for patients identified as low-risk MSK using the Keele STarT MSK Tool, which classifies patients as low risk (0–4), medium risk (5–8), or high risk (9–12) [13,14]. It operationalizes the STarT MSK recommendation for low-risk patients [12,17], which emphasizes education, supported self-management, and maintenance of physical activity, and draws on formative qualitative research to ensure cultural relevance and acceptability in the Saudi healthcare context [27]. Rather than functioning as a general digital self-management resource, the program was designed as a structured, risk-matched intervention tailored to the needs of low-risk patients. The program was designed based on social cognitive theory [32]. It integrated behavior-change strategies to enhance patients’ self-efficacy and support independent management through structured goal setting, progressive exercise, regular feedback, and supported self-management delivered in a patient-centered manner. This hybrid model accommodated patients’ preference for an initial in-person consultation before transitioning to virtual follow-up, while also addressing the practical challenges of providing resource-intensive in-person physiotherapy to the large number of low-risk patients identified in the primary care center. The bundled care pathway included one in-person physiotherapy assessment in Week 1, followed by five weekly 20-min virtual follow-up consultations over the next five weeks through the Physiotherapy Telerehabilitation Center at PSMMC using the ANAT telemedicine platform. Sehhatek automatically delivered appointment notifications. During the initial in-person consultation, participants underwent a comprehensive physiotherapy assessment, received individualized education, established functional goals, and were prescribed a condition-specific home exercise program. Participants must complete baseline outcome measures before the first virtual follow-up consultation. The initial in-person session lasted 45–60 min. Across the six weeks, sessions followed a common progression: assessment and foundation (week 1), pain modulation and mobility (week 2), activation and load (week 3), endurance and functional control (week 4), functional integration (week 5), and review and transition at discharge (week 6). Condition-specific exercise modules for back, neck, shoulder, knee, and multisite pain followed the same progression of mobility, activation, strength, endurance, functional integration, and maintenance, with condition-appropriate exercises and education topics. Subsequent virtual sessions focused on reviewing symptoms and functional progress, progressing home exercises, reinforcing self-management strategies, addressing barriers to adherence, and monitoring for clinical deterioration. The six-contact schedule was established before recruitment as a pragmatic service-delivery model rather than as an empirically determined minimum treatment dose. It was intended to allow weekly exercise progression, reinforce self-management, review adherence barriers, and support clinical monitoring while remaining feasible within local service capacity. The hybrid sequence was also informed by formative qualitative findings indicating that patients preferred an initial in-person consultation followed by virtual support [27]. The study was not designed to determine the optimal number or frequency of contacts or to evaluate a dose–response relationship. Participants were advised to perform their prescribed home exercise program for 15–25 min per day, on at least four days each week. They had continuous access to supplementary educational materials provided in the first session. Detailed intervention protocol materials, including condition-specific exercise modules and educational content, are available from the corresponding author upon reasonable request. Four trained physiotherapists supervised by the principal investigator delivered the intervention. Clinical attendance records were used to monitor program adherence. Participants were considered to have completed the program if they attended the initial in-person consultation and at least three of the five scheduled virtual follow-up sessions (a minimum of four completed contacts). Participants who reported an increase in pain of more than two points on the Numeric Pain Rating Scale or developed new neurological symptoms during follow-up were referred for an in-person physiotherapy review in accordance with the predefined escalation protocol defined specifically for this study. This 2-point threshold reflects the established minimal clinically important difference for the Numeric Pain Rating Scale [33].

### 2.8. Outcomes

The prespecified primary longitudinal outcomes were pain intensity, measured using the Numeric Pain Rating Scale (NPRS; 0–10) [33]; MSK health and function, measured using the Musculoskeletal Health Questionnaire (MSK-HQ; 0–56) [34,35]. Psychosocial prognostic risk, measured using the Örebro Musculoskeletal Pain Questionnaire (ÖMPQ; 0–210) [36,37], was a prespecified secondary longitudinal outcome. We assessed all three outcomes at baseline, 6 weeks, and 12 weeks and analyzed them using the same mixed-effects modeling framework. ÖMPQ total scores were calculated according to the questionnaire’s scoring instructions. To maximize follow-up, we sent participants who had not completed an assessment three reminder messages at different times, followed by a telephone call and, after a one-day interval, a final message. Participants who did not complete the 6-week assessment after this sequence were not approached again for the 12-week assessment. Patient satisfaction, assessed at 12 weeks using a 5-point Likert scale (very dissatisfied, dissatisfied, neither, satisfied, very satisfied), was a secondary outcome; the STarT MSK trial used a comparable 5-category ordinal satisfaction measure [17]. The Global Rating of Change (GROC; 11-point scale, −5 to +5, anchored from “much worse” at −5 to “completely recovered” at +5, with 0 indicating “no change”) [38] was analyzed as an exploratory outcome. We used all Arabic versions with formal permission from the respective copyright holders. A license to obtain both the self-report and clinician-completed versions of the tool is available on request (https://www.keele.ac.uk/startmsk/ (accessed on 1 May 2025)).

### 2.9. Statistical Analysis

Analyses were conducted in IBM SPSS Statistics version 31 (IBM Corp., Armonk, NY, USA), with a two-tailed significance threshold of *p* < 0.05. Baseline characteristics were summarized descriptively for each cohort. Continuous variables were compared using the independent-samples *t*-test when normality was met in both cohorts, assessed by the Shapiro–Wilk test with graphical inspection, and using the Mann–Whitney U test otherwise; categorical variables were compared using the Pearson chi-square test or Fisher’s exact test when any expected cell count was below five. Effect sizes are reported alongside *p*-values. Because a sequential cohort design does not produce equivalent groups, baseline differences are described and addressed through sensitivity analysis rather than treated as evidence of comparability.

Changes in NPRS, MSK-HQ, and ÖMPQ at baseline, 6 weeks, and 12 weeks were analyzed using Linear Mixed-Effects Models (LMMs) estimated by restricted maximum likelihood. Each model included fixed effects for cohort, time (treated as a categorical factor), and the cohort-by-time interaction. Each model also included a random intercept for participant and a random slope for time, which was retained because likelihood ratio testing showed improved fit for all three outcomes (Supplementary Material S1). The primary models included no covariates. Estimated marginal means and contemporaneous between-cohort differences were calculated at baseline, 6 weeks, and 12 weeks. Differential-change contrasts derived from the cohort-by-time interaction estimated whether change from baseline differed between cohorts at each follow-up. These represent distinct estimands: the contemporaneous difference compares cohorts at an individual time point, whereas the differential change compares their changes from baseline. This difference-in-change comparison accounts for the baseline outcome level observed in the model. However, it does not eliminate confounding that arises from the non-randomized sequential cohort design. Mixed-effects models use all available observations under a missing-at-random assumption. No adjustment for multiple testing across the three longitudinal outcomes was applied; consequently, *p*-values were interpreted alongside effect estimates, 95% confidence intervals, and the consistency of findings across outcomes. Because the ÖMPQ also includes pain and function items, concordance across the three instruments was not treated as independent evidence.

Model assumptions were evaluated separately for each outcome by examining residual distributions, residual-versus-predicted plots, and the influence of outlying observations. Sensitivity analyses examined adjustments for baseline characteristics, alternative random-effects specifications, exclusion of observations with standardized residuals greater than 3 standard deviations, and restriction to participants with complete data at all 3 time points. To address joint confounding, we additionally fitted four models for each outcome (SPSS MIXED, REML, subject = participant; Models A and B had a random intercept and time slope with unstructured covariance, and Models C and D a random intercept only). Model A added age (centered), gender, referral pain site, previous physiotherapy, and symptom duration (centered) simultaneously as main effects to the primary model. Model B additionally included the interaction of each of these covariates with time, allowing recovery trajectories to differ by covariate. Model C used the 6- and 12-week observations only and included the centered baseline outcome score and its interaction with time (random intercept), yielding a baseline-severity-adjusted 12-week between-cohort difference. Model D combined baseline outcome score, age, gender, referral pain site, previous physiotherapy, and symptom duration, each with its interaction with time, in the 6- and 12-week data (random intercept). Covariates were selected on clinical grounds as plausible determinants of the outcomes, and their number was limited by the sample size. Further details are provided in Supplementary Material S1, and the study workflow is shown in Figure S1. Post hoc exploratory analyses examined whether the differential change differed by sex and by referral pain site (back, knee, and shoulder; the neck, multisite, and other categories had too few participants) by fitting the primary model within each subgroup and testing the cohort × time × subgroup interaction. A per-protocol analysis excluded stratified-care participants who did not complete the program, defined as attendance at the initial in-person assessment and at least three of the five scheduled virtual follow-up sessions. These analyses are reported in Section 3.6 and Supplementary Material S1. Multiple imputation was also conducted as a sensitivity analysis: 30 imputed datasets were generated by chained-equations regression imputation of missing 6- and 12-week outcome values, using baseline outcome scores, cohort, age, gender, nationality, symptom duration, body mass index, referral pain site, and prior physiotherapy as predictors; the primary mixed-effects model was refitted to each imputed dataset, and cohort-by-time estimates were pooled using Rubin’s rules. To examine departures from the missing-at-random assumption, we also conducted a delta-adjusted (tipping-point) multiple-imputation analysis: missing 6- and 12-week values were imputed in 30 datasets from regression models including baseline outcome score, cohort, age, gender, referral pain site, previous physiotherapy, and symptom duration (and, for 12-week values, the 6-week value); a fixed shift (δ) in the unfavorable direction was then added to the imputed values of the stratified-care cohort only, the primary mixed-effects model was refitted, and estimates were pooled using Rubin’s rules. δ was increased until the 12-week cohort-by-time contrast no longer excluded zero. For the responder analyses, we additionally repeated the comparisons counting participants with missing 12-week data as non-responders. The delta-adjusted multiple-imputation and non-responder-imputation sensitivity analyses were conducted in Python (version 3.11; statsmodels version 0.15.0); all other analyses were conducted in SPSS. GROC was analyzed as an exploratory outcome because it was not designated as a primary or secondary outcome. Responder analyses were conducted for NPRS and MSK-HQ to quantify the proportion of participants achieving a clinically meaningful change at 12 weeks; no responder threshold was specified for ÖMPQ. Response on the NPRS was defined as a reduction of at least 2 points. For MSK-HQ, two thresholds were examined and reported: an increase of at least 6 points, operationalizing the 5.5-point minimal important change reported by Price et al. [39], and an increase of at least 6.83 points, corresponding to the Arabic-specific minimal detectable change at the 95% confidence level reported by Algarni et al. [35]; the latter reflects measurement error rather than clinical importance and is reported to test whether conclusions depend on the threshold used. Responder proportions were compared between cohorts using the Pearson chi-square test, with risk differences, risk ratios, and odds ratios reported alongside 95% confidence intervals.

### 2.10. Ethical Approval

The PSMMC Institutional Review Board (IRB) approved the study (E-2557, 30 April 2025). The study is reported in accordance with the Strengthening the Reporting of Observational Studies in Epidemiology (STROBE) guidelines for cohort studies [40] (see the STROBE checklist). All participants provided written informed consent. The study was retrospectively registered with the Research Registry on 19 August 2026 (UIN: researchregistry12009). The study was conducted as part of a thesis and followed the proposal approved by the IRB; departures from the proposal are listed in Supplementary Material S1 (Table S4). Cohort allocation was determined by the study design rather than by independent service changes. The Global Rating of Change was not part of the approved proposal and is analyzed as an exploratory outcome. Data were anonymized, stored on encrypted systems, and retained for five years in accordance with Saudi data protection regulations.

## 3. Results

### 3.1. Participant Flow and Baseline Characteristics

**Figure 2:**
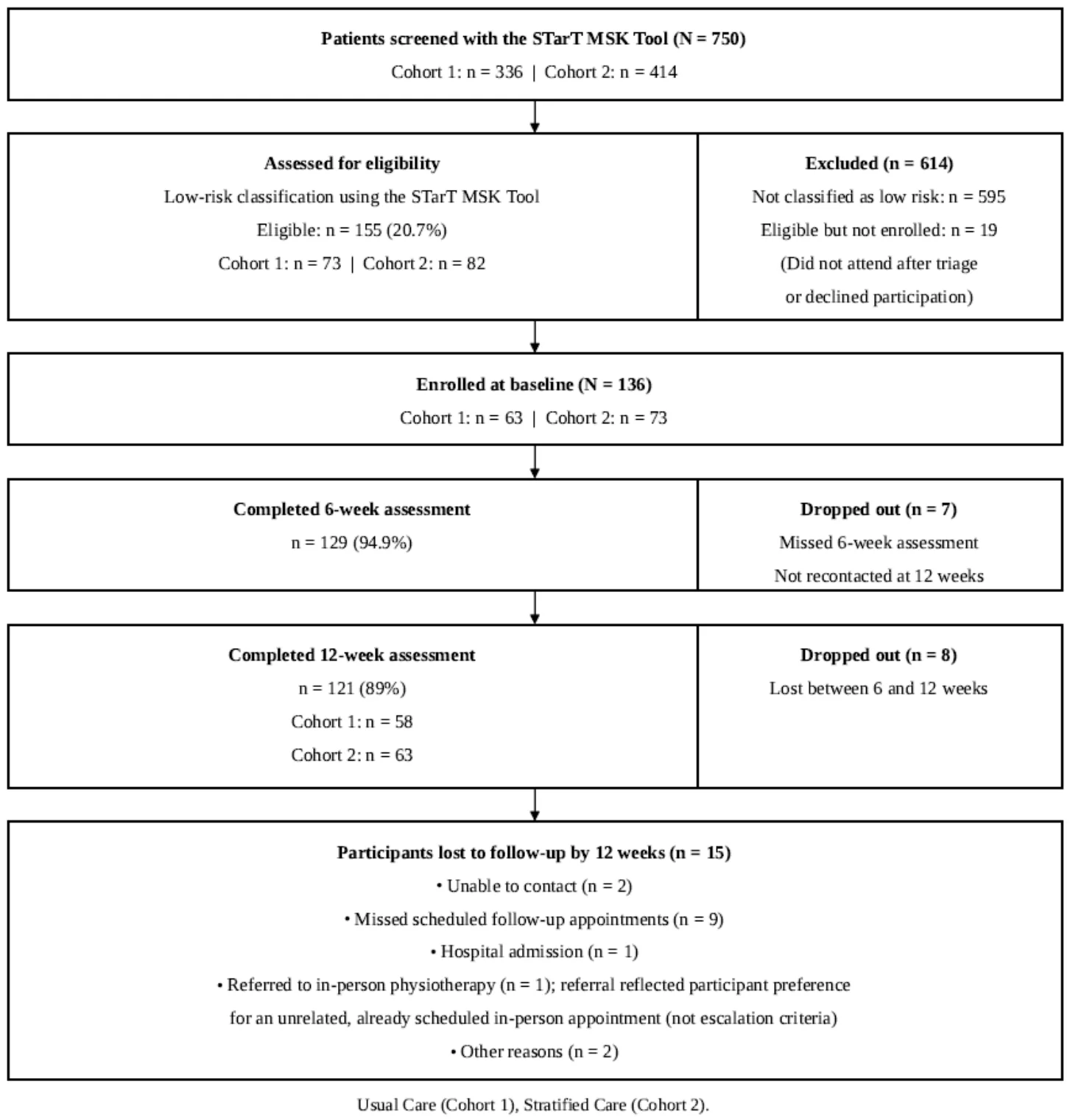
Participant flow through screening, enrollment, and follow-up. Of 750 patients screened using the Keele STarT MSK Tool, 155 were classified as low risk, and 136 were enrolled: 63 in the usual-care cohort and 73 in the stratified-care cohort. Follow-up assessments were completed by 129 participants at 6 weeks and 121 at 12 weeks. Participants who missed the 6-week assessment were not recontacted at 12 weeks. The figure presents reasons for incomplete follow-up.

Of 750 screened patients, 155 (20.7%) were low-risk and eligible, and 136 were enrolled (63 in usual care, 73 in stratified care). Outcome data were available for 129 participants (94.9%) at 6 weeks and for 121 (89.0%) at 12 weeks. Retention at 12 weeks was 92.1% in usual care and 86.3% in stratified care (Pearson χ^2^ *p* = 0.285). Figure 2 shows reasons for incomplete follow-up and the complete participant flow. Reasons for missing 12-week data in the usual-care cohort (n = 5) were inability to reach the participant (n = 2), hospital admission (n = 1; reason unknown; withdrawn from follow-up), reporting being too unwell to complete the telephone follow-up (n = 1), and not completing the data despite reminders (n = 1). In the stratified-care cohort (n = 10), the reasons were referral to face-to-face physiotherapy (n = 1); no follow-up session attended despite three contact attempts, followed by discharge (n = 3); attendance at only one follow-up session, followed by discharge (n = 1); not completing the 12-week data despite reminders (n = 1); and completing the program sessions but not the 12-week assessment despite three reminders (n = 4). Seven participants (two usual care, five stratified care) missed the 6-week assessment, and none of them provided 12-week data.

During the usual-care index consultation, participants could receive more than one management decision; therefore, the categories were not mutually exclusive, and their frequencies exceeded the total number of participants. Of the 63 usual-care participants, 49 (77.8%) had medication prescribed (the medication type was not recorded), 34 (54.0%) underwent or were referred for imaging, 15 (23.8%) were referred for conventional physiotherapy, and 11 (17.5%) were referred to a secondary-care specialist. Advice or education, including lifestyle modification, was not a predefined field and was entered as free text under “other decision” for 15 participants (23.8%); this is therefore likely an underestimate. These findings reflect physician management decisions recorded at the index consultation; we did not verify subsequent receipt, frequency, or content of care.

All 73 participants in the stratified-care cohort received an official physiotherapy referral, were enrolled in the eSELF-MSK program, and attended the initial in-person assessment. Participants attended a median of 5 program contacts (IQR 5–6; range 1–6), and 68 of 73 (93.2%) attended the initial assessment and at least three follow-up sessions. Five participants (6.8%) did not meet this threshold. The cohorts were similar in body mass index, symptom duration, previous pain episodes, previous surgery, and baseline MSK-HQ and ÖMPQ scores. Participants in the stratified care cohort were older, reported higher baseline pain, had marginally higher STarT MSK scores within the low-risk range, were less likely to have received previous physiotherapy, and had a different distribution of referral pain sites (Table 1). No stratified-care participant was referred for in-person review under the escalation protocol during the program, and no usual-care participant was changed to an in-person session because of deterioration. However, the escalation criteria were not monitored by a structured procedure in either cohort beyond clinical contacts and review of outcome data. In the outcome data, NPRS had increased by more than 2 points from baseline at 6 or 12 weeks in 10 of 61 usual-care participants and in 1 of 68 stratified-care participants with follow-up pain data (the latter at 12 weeks, after the six-week program had ended), so the absence of recorded escalation should not be interpreted as the absence of clinically important deterioration. The one participant referred to in-person physiotherapy (Figure 2) did so for reasons unrelated to the escalation criteria.

**Table 1:** Baseline demographic and clinical characteristics by study cohort, N = 136.

Characteristic	Usual Care (n = 63)	Stratified Care (n = 73)	*p*	Effect Size
**Demographic**				
Age, y—median (IQR)	36.0 (28.0–44.0)	43.0 (34.0–51.0)	0.005 *	r = 0.28
Female sex—n (%)	31 (49.2)	46 (63.0)	0.105	V = 0.14
Saudi nationality—n (%)	60 (95.2)	73 (100.0)	0.097	V = 0.16
BMI, kg/m^2^—median (IQR)	27.2 (23.6–31.2)	27.7 (24.7–31.2)	0.630	r = 0.05
**Clinical**				
**Referral pain site—n (%)**			0.001 *	V = 0.35
Back	29 (46.0)	25 (34.2)		
Knee	12 (19.0)	29 (39.7)		
Shoulder	7 (11.1)	15 (20.5)		
Neck	9 (14.3)	1 (1.4)		
Multisite	6 (9.5)	3 (4.1)		
**Symptom duration—n (%)**			0.915	V = 0.06
<2 weeks	6 (9.5)	7 (9.6)		
2–4 weeks	7 (11.1)	8 (11.0)		
1–3 months	10 (15.9)	15 (20.5)		
>3 months	40 (63.5)	43 (58.9)		
Previous MSK pain episode—n (%)	24 (38.1)	35 (47.9)	0.248	V = 0.10
Previous surgery—n (%)	3 (4.8)	1 (1.4)	0.336	V = 0.10
Previous physiotherapy—n (%)	15 (23.8)	7 (9.6)	0.025 *	V = 0.19
**Baseline outcome measures**				
NPRS (0–10)—median (IQR)	4.0 (3.0–5.0)	5.0 (4.0–6.0)	0.002 *	r = 0.30
STarT MSK total score (low-risk range 0–4)—median (IQR)	3.0 (2.0–4.0)	4.0 (3.0–4.0)	0.005 *	r = 0.25
MSK-HQ (0–56)—mean (SD)	39.4 (8.8)	37.5 (8.5)	0.190	d = −0.23
ÖMPQ (0–210)—mean (SD)	77.8 (28.2)	84.7 (27.3)	0.148	d = 0.25

* *p* < 0.05. Continuous variables were compared using the independent-samples *t* test (Cohen d; Levene test non-significant for both variables) when normally distributed, or the Mann–Whitney U test (rank-biserial r) otherwise; categorical variables were compared using the Pearson chi-square test, or the Fisher exact test where any expected cell count was below five (Cramér V in both cases). Each effect size corresponds to the test applied: Cohen d is reported only for mean-based comparisons and rank-biserial r only for rank-based comparisons. Benchmarks: Cohen d 0.20 small, 0.50 medium, 0.80 large; rank-biserial r 0.10 small, 0.30 medium, 0.50 large; Cramér V 0.10 small, 0.30 medium, 0.50 large. Effect sizes are oriented as stratified care relative to usual care. Abbreviations: SD, standard deviation; IQR, interquartile range; BMI, body mass index; NPRS, Numeric Pain Rating Scale; MSK-HQ, Musculoskeletal Health Questionnaire; ÖMPQ, Örebro Musculoskeletal Pain Questionnaire. No participant scored 0 on the STarT MSK Tool; the observed range was 1–4. Baseline *p*-values in Section 3.2 and Table 2 are model-based estimates and may differ slightly from these unadjusted tests.

**Table 2:** Estimated marginal means and cohort-by-time contrasts (usual care, n = 63; stratified care, n = 73).

Time Point	Usual Care, Mean (95% CI)	Stratified Care, Mean (95% CI)	Between-Group Difference (95% CI)	*p*	Differential Change from Baseline (95% CI)	*p*
**NPRS (0–10)**						
Baseline	4.11 (3.68–4.54)	5.00 (4.60–5.40)	+0.89 (+0.31 to +1.47)	0.003	—	—
6 weeks	3.85 (3.34–4.36)	2.40 (1.91–2.88)	−1.45 (−2.16 to −0.75)	<0.001	−2.34 (−3.05 to −1.64)	<0.001
12 weeks	3.74 (3.11–4.38)	2.51 (1.91–3.12)	−1.23 (−2.11 to −0.35)	0.007	−2.12 (−2.95 to −1.29)	<0.001
**MSK-HQ (0–56)**						
Baseline	39.41 (37.32–41.51)	37.47 (35.52–39.41)	−1.95 (−4.81 to +0.91)	0.181	—	—
6 weeks	40.83 (38.62–43.03)	46.18 (44.11–48.26)	+5.36 (+2.33 to +8.39)	<0.001	+7.31 (+4.49 to +10.12)	<0.001
12 weeks	40.83 (38.27–43.39)	45.96 (43.53–48.40)	+5.14 (+1.60 to +8.67)	0.005	+7.08 (+3.70 to +10.47)	<0.001
**ÖMPQ (0–210)**						
Baseline	77.78 (70.74–84.82)	84.71 (78.17–91.25)	+6.94 (−2.67 to +16.54)	0.156	—	—
6 weeks	77.11 (69.74–84.49)	60.24 (53.32–67.16)	−16.88 (−26.99 to −6.77)	0.001	−23.81 (−31.81 to −15.82)	<0.001
12 weeks	78.46 (69.97–86.94)	57.56 (49.52–65.59)	−20.90 (−32.59 to −9.21)	<0.001	−27.83 (−37.90 to −17.76)	<0.001

### 3.2. Longitudinal Outcomes

The cohort by-time interaction was statistically significant for all three outcomes: NPRS, F(2, 162.9) = 22.82; MSK-HQ, F(2, 164.1) = 14.17; ÖMPQ, F(2, 157.1) = 20.18; all *p* < 0.001. At baseline, in the mixed model, the stratified-care cohort had higher NPRS scores than the usual-care cohort (*p* = 0.003), whereas baseline MSK-HQ (*p* = 0.181) and ÖMPQ (*p* = 0.156) scores did not differ significantly (unadjusted baseline tests are in Table 1). Contemporaneous between-cohort comparisons and differential changes from baseline are reported separately in Table 2.

Estimates are from linear mixed-effects models with fixed effects for cohort, categorical time, and their interaction, and participant-specific random intercept and slope. Between-cohort difference is stratified care minus usual care at that time point. Negative values favor stratified care for NPRS and ÖMPQ, whereas positive values favor stratified care for MSK-HQ. Differential change is the cohort-by-time contrast. The first *p* column tests the between-cohort difference at each time point; the second *p* column tests the differential change at 6 and 12 weeks.

### 3.3. Responder Analyses

Because group mean differences do not indicate how many individual participants improved meaningfully, the proportion of participants achieving a defined change threshold was compared between cohorts at 12 weeks (Table 3). In the Methods, Statistical Analysis section, we provide threshold definitions and citations. Responder proportions were higher in the stratified cohort (risk ratios 1.8–2.6) across all definitions, and the two MSK-HQ thresholds produced closely similar estimates.

All comparisons by Pearson chi-square: NPRS χ^2^(1) = 21.75, *p* < 0.001; MSK-HQ ≥ 6 points χ^2^(1) = 9.09, *p* = 0.003; MSK-HQ ≥ 6.83 points χ^2^(1) = 9.21, *p* = 0.002. No expected count fell below five in any analysis. All three responder analyses are post hoc; the thresholds were taken from the literature. The 6-point threshold operationalizes the 5.5-point minimal important change reported by Price et al. [39]; the 6.83-point threshold is the Arabic-specific minimal detectable change at the 95% confidence level reported by Algarni et al. [35] and reflects measurement error rather than clinical importance. Counts include participants with 12-week data (usual care n = 58; stratified care n = 63); the analysis counting participants with missing 12-week data as non-responders is in Table S1 of Supplementary Material S1.

### 3.4. Patient-Reported Experience

Satisfaction with care and the GROC were assessed at 12 weeks and are presented as complete ordinal distributions in Table 4. Satisfaction was a prespecified secondary outcome. In the stratified-care cohort, three participants reported no change, and three reported worsening in the GROC. None were referred under the escalation protocol; however, NPRS had increased by more than 2 points at 12 weeks in one of the three (see Section 3.1).

Comparisons were conducted using the Mann–Whitney U test. Global Rating of Change (GROC) was exploratory, and the threshold of ≥+3 was selected post hoc. Ten usual-care participants (17.2%) and three stratified-care participants (4.8%) reported GROC scores of −5 to −1, indicating perceived worsening, consistent with the pattern of between-group differences observed in the continuous outcome measures. No participant was escalated for deterioration, but NPRS increased by more than 2 points at 12 weeks in one of the ten usual-care participants and one of the three stratified-care participants who reported worsening, so perceived worsening may reflect clinically important deterioration in some cases. GROC captures a broader, patient-reported sense of overall change, which may not align exactly with pain-specific measures.

### 3.5. Sensitivity Analyses

The primary mixed-effects models assume that missing data are unrelated to unobserved outcome values. To check this assumption, we compared baseline characteristics of the 121 participants who completed the 12-week follow-up with the 15 who did not. Non-completers were somewhat older, reported greater baseline pain, and were more often enrolled in the stratified-care cohort (standardized differences all exceeding 0.20). However, this comparison is underpowered to draw firm conclusions. Sensitivity analyses produced estimates consistent with those from the primary mixed-effects models (Table 5). Adjusting for referral pain site, age, and previous physiotherapy care; using an alternative random-effects specification; excluding observations with standardized residuals exceeding three standard deviations; and restricting the sample to the 121 participants with complete observations at all three time points did not materially alter the estimated between-cohort differences in change. A post hoc ANCOVA examining baseline-adjusted between-cohort differences at 12 weeks also produced findings in the same direction. However, this analysis estimated a different quantity than the cohort-by-time contrast.

Multiple imputation using 30 imputed datasets produced pooled 12-week cohort-by-time contrasts of −2.01 points for NPRS (95% CI −2.83 to −1.20), +6.80 points for MSK-HQ (95% CI +3.37 to +10.23), and −27.39 points for ÖMPQ (95% CI −37.32 to −17.47). In the jointly adjusted models (Table 5, Models A–D), the between-cohort differences remained in the same direction and statistically significant for all three outcomes; Table S2 of Supplementary Material S1 shows the contemporaneous 12-week differences and the effect of each adjustment. In delta-adjusted multiple imputation, the stratified-care advantage remained statistically significant when missing follow-up values in the stratified-care cohort were assumed to be substantially worse than predicted (by up to 5 points for NPRS, 16 points for MSK-HQ, and 60 points for ÖMPQ; significance was lost at shifts of 6, 20, and 80 points, equivalent to approximately 2.3 to 3.4 baseline standard deviations), and when participants with missing 12-week data were counted as non-responders in the responder analyses (Table S1 of Supplementary Material S1). Estimates from the other sensitivity analyses in Table 5 remained statistically significant (*p* < 0.001). Together, these analyses show that the findings held up across covariate adjustments, model specifications, outlier handling, and missing-data methods examined. However, they cannot rule out residual or unmeasured confounding, which is inherent to the non-randomized sequential cohort design. Calendar period remains inseparable from cohort assignment, so none of these adjustments can address temporal or seasonal confounding.

Values are between-cohort estimates at 12 weeks with 95% confidence intervals. The primary model, the first six sensitivity analyses, and Models A and B report the cohort-by-time contrast at 12 weeks, representing the between-cohort difference in change from baseline. The post hoc ANCOVA reports the baseline-adjusted between-cohort mean difference at 12 weeks and therefore represents a different estimand. Models C and D likewise report baseline-adjusted 12-week mean differences (6- and 12-week observations only); Model D additionally includes the joint covariates and their interactions with time. Models A and B report the cohort-by-time contrast with joint adjustment for age, gender, referral pain site, previous physiotherapy, and symptom duration; Model B also includes covariate-by-time interactions. The multiple-imputation analysis reports the pooled 12-week cohort-by-time contrast across 30 imputed datasets using Rubin’s rules. All estimates remained statistically significant (*p* < 0.001). Among the repeated-measures model specifications, excluding Models A and B, ANCOVA, and multiple imputation, the maximum deviation from the primary estimate was 0.09 points for NPRS, 0.41 points for MSK-HQ, and 0.16 points for ÖMPQ. Models A and B deviated from the primary estimate by at most 0.03 points for NPRS, 0.56 points for MSK-HQ, and 0.71 points for ÖMPQ. The outlier-exclusion analysis removed 3 observations for NPRS, 2 for MSK-HQ, and 1 for ÖMPQ from the 386 available observations. The complete-case analysis included 363 observations from 121 participants. ANCOVA included the 121 participants with baseline and 12-week observations, whereas multiple imputation included all 136 participants.

### 3.6. Exploratory Subgroup and Per-Protocol Analyses

These post hoc analyses are summarized in Table S3 of Supplementary Material S1. They are underpowered and should be interpreted as hypothesis-generating rather than confirmatory. By sex (59 men and 77 women), the differential change favored stratified care on all three outcomes in both men and women, and there was no evidence that the effect differed by sex (cohort × time × sex interaction *p* = 0.61 for ÖMPQ, 0.55 for MSK-HQ, and 0.53 for NPRS). Among participants referred for back (n = 54), knee (n = 41), or shoulder (n = 22) pain, the estimates also favored stratified care, with the smallest effects in back pain; the 95% confidence intervals included zero for the MSK-HQ in back pain (+3.64, 95% CI −1.81 to +9.09) and for the NPRS in knee pain (−1.17, 95% CI −2.42 to +0.08). The NPRS site-specific models used a random intercept only because the model with a random slope did not converge. The interaction tests did not show statistically significant heterogeneity across the three sites (*p* = 0.26 for ÖMPQ, 0.09 for MSK-HQ, and 0.34 for NPRS), but these tests have little power, and the neck (n = 10) and multisite (n = 9) groups were too small to analyze. Subgroup estimates, particularly for shoulder pain (7 usual-care and 15 stratified-care participants), are imprecise. In the per-protocol analysis, 68 of 73 stratified-care participants (93.2%) completed the program, defined as attendance at the initial in-person assessment and at least three of the five scheduled virtual sessions. Excluding the five non-completers gave estimates essentially unchanged from the primary analysis (differential change at 12 weeks: ÖMPQ −27.75, NPRS −2.14, MSK-HQ +7.10; all *p* < 0.001). Excluding non-completers may introduce selection bias, so this analysis is supportive rather than causal.

### 3.7. Summary of Findings

The model-estimated differential change favored the stratified-care cohort by 2.34 points in pain intensity at 6 weeks and 2.12 points at 12 weeks; by 7.31 and 7.08 points on the MSK-HQ; and by 23.81 and 27.83 points on the ÖMPQ. At 12 weeks, 68.3% versus 25.9% achieved at least a 2-point reduction in pain intensity, and 88.9% versus 51.7% reported satisfaction with care. Estimates were similar in direction and statistical significance after adjustment for baseline imbalances, exclusion of outlying observations, and alternative random-effects specifications. Because the design was a non-randomized sequential comparison and all outcomes were self-reported by unblinded participants, expectation effects cannot be excluded as contributors to the observed differences, and this is addressed in the Discussion.

## 4. Discussion

### 4.1. Principal Findings

In this sequential cohort study of 136 patients classified as low risk, implementation of a bundled STarT MSK-guided care pathway was associated with greater improvement than usual care across all three outcome measures. The pathway combined communication of risk classification, clinician training, an initial in-person physiotherapy assessment, individualized education and exercise, supported self-management, and five virtual follow-up consultations. Because both cohorts underwent STarT MSK screening and all enrolled participants were classified as low risk, the observed differences cannot be attributed to risk screening, the STarT MSK Tool, or any other pathway component. Compared with usual care, the stratified-care cohort showed additional model-estimated improvements of 2.34 NPRS points and 7.31 MSK-HQ points at 6 weeks, with similar differential changes at 12 weeks (2.12 and 7.08 points). Thresholds for clinically important change in an individual are not thresholds for between-cohort effects, and the NPRS estimates at 12 weeks differ by estimate: −2.12 for the differential change, −1.23 for the contemporaneous difference, and −1.67 for the baseline-adjusted ANCOVA difference. The 95% confidence interval of the differential change (−2.95 to −1.29) includes benefits smaller than 2 points, so these data do not show that the average benefit exceeds that threshold. Differential reductions in ÖMPQ scores favored stratified care: 23.81 points at 6 weeks and 27.83 points at 12 weeks. Approximately two-thirds of participants in the stratified-care cohort achieved a clinically important reduction in pain, compared with one-quarter in the usual-care cohort, and 88.9% reported satisfaction in the stratified-care cohort, compared with 51.7% in the usual-care cohort. Estimates were similar in direction and statistical significance after adjustment for baseline imbalances, alternative random-effects specifications, exclusion of outlying observations, and restriction to participants with complete follow-up. Notably, the estimated means for the usual-care cohort remained similar at the 12-week follow-up across all three instruments, in contrast to the improvement observed in the stratified-care cohort (Table 2). Participant engagement with the intervention was high, with 93.2% meeting the operational definition of program completion.

### 4.2. Comparison with Existing Evidence

The UK STarT MSK trial evaluated a primary-care decision-support [17] pathway, whereas the Dutch SBPA trial evaluated STarT MSK–matched blended physiotherapy [41]. Neither reported a statistically significant between-group clinical effect, although the Dutch pilot was substantially underpowered. By contrast, sequential-cohort studies that, like the present study, delivered a structured treatment program found at least one significant patient-reported effect: Gomes et al. [20] on pain and Foster et al. [19] on disability, though not pain specifically. The present study’s result is consistent with this second group in direction and significance; however, its magnitude is larger, which we examine below (Table 6).

Hill et al. [17] evaluated the same stratification tool across 24 UK general practices and found no significant difference in time-averaged pain intensity over 6 months (adjusted mean difference −0.16; 95% CI, −0.65 to 0.34) or in most secondary outcomes, including MSK health and quality of life. Earlier implementation studies reported smaller effects than the present findings: Foster et al. [19] reported a non-significant pain difference of 0.29 points, and Gomes et al. [20] reported a significant difference of 0.88 points). Meta-analytic estimates for digital self-management in MSK pain indicate small effects on pain (SMD 0.19, 95% CI 0.06–0.31) and disability (0.14, 95% CI 0.03–0.25) [43]. This trial is part of the broader STarT MSK research program, which also included the tool development cohort (n = 1890) and an earlier pilot trial (n = 524) [31]. We cite Hill et al. [17], the peer-reviewed primary publication reporting the results of the cluster-randomized trial, rather than the more recent Foster et al. [31] program report, which covers all four STarT MSK work packages but presents the same trial data as Hill et al. [17] for the outcomes discussed here.

Beyond the original UK STarT MSK research program, only one other independent intervention trial of the Keele STarT MSK Tool has been published: a Dutch cluster-randomized trial testing it within a matched-treatment, blended-care physiotherapy model for neck and shoulder pain, which also found no significant difference over 9 months (adjusted mean difference −0.91; 95% CI, −7.39 to 5.56). Although recruitment was substantially below target, the authors identified potential selection bias [41]. To our knowledge, Hill et al. [17] and Van Tilburg et al. [41] are the two published randomized evaluations of STarT MSK–guided care, with Van Tilburg et al. representing the only independent trial outside the original UK research program. This narrow evidence base—two null RCTs and a handful of sequential-cohort implementations—is itself a limitation of the comparator literature, not only of the present study. Stratified care guided by this tool is relatively new: the Keele STarT MSK Tool was validated in 2021 [14], and its first cluster-randomized trial was published in 2022 [17]. This pattern aligns with a broader recognition that between-study heterogeneity in stratified-care trial results reflects identifiable study-level factors rather than random variation [45]. Complementary evidence has examined the tool’s prognostic performance rather than the clinical effectiveness of stratified care. In a multicenter United States outpatient physical therapy cohort, Beneciuk et al. found evidence of construct and longitudinal predictive validity across common musculoskeletal pain presentations. The STarT MSK Tool explained additional variability in pain intensity, pain interference, and physical health scores at two and six months [16]. These findings support the tool’s prognostic relevance in outpatient physical therapy, but they do not establish that using the tool to allocate matched treatment improves clinical outcomes. The present study addressed a different question: it evaluated outcomes after implementing a bundled care pathway among patients already classified as low risk and did not assess prognostic discrimination across risk categories.

We may relate the large observed effect to several factors. First, the intervention tested differs. The STarT MSK trial evaluated a one-off decision-support prompt: general practitioners received the risk tool and a menu of treatment options, but the clinician and patient made the subsequent treatment decision, so matched care was not guaranteed [17]. By contrast, the present study delivered a structured program through scheduled in-person and virtual contacts, which 93.2% of participants completed. This difference in intervention delivery may have contributed to the larger observed estimate, although it cannot be separated from differences in design, population, comparator care, or risk of bias. Consistent with Gomes et al. [20] who attributed their larger effect over Foster et al. [19] to the same distinction: physiotherapist-delivered treatment with high adherence, rather than clinician prompting alone. A similar contrast is evident in a recent Australian feasibility trial of STarT MSK–guided stratified telehealth care, where adherence to the model of care was uncertain because of low appointment attendance in both study arms; the present study’s 93.2% program completion suggests a different level of engagement with a structured, delivered intervention [26]. This explanation cannot be tested directly because the studies differ in design, population, and comparator.

Second, the comparator may have performed poorly in our setting, inflating the apparent benefit of stratified care without necessarily indicating a more effective intervention. UK national guidance recommends advice, education, reassurance, and self-management as first-line care for low-risk patients with back pain [46], so in UK primary care the incremental benefit of a stratified pathway over usual care may be small; whether Saudi usual care matched this standard is unclear. A related pre-implementation analysis found low concordance between physician management decisions and STarT MSK recommendations, overall (22.3%) and among low-risk patients specifically (30.1%), with discordance driven mainly by over-investigation rather than by education, reassurance, and supported self-management [10]. This is consistent with, though it does not prove, a lower-performing comparator: outcomes in the usual-care cohort did not improve on any of the three instruments over 12 weeks, consistent with low concordance in the comparator. We present this as a plausible contributing factor rather than an established explanation.

Third, the population is narrower. The STarT MSK trial recruited across all three risk strata and was explicitly not powered to detect differences within subgroups. Digital self-management interventions have produced small average benefits in broader MSK populations [43], although evidence specific to patients classified as low risk by STarT MSK remains limited. One possible explanation is that outcomes associated with a low-risk pathway, delivered with high participant attendance, may differ from effects averaged across populations comprising multiple risk groups. Medium- and high-risk patients may also require more complex interventions that are more challenging to implement consistently. However, this interpretation is hypothesis-generating only. The present study included only low-risk patients and was not designed to determine whether risk-group composition, intervention complexity, or adherence explains differences between studies.

Fourth, and most importantly, we cannot exclude design-related bias. Participants in this non-randomized, unblinded design knew which care they received, and all outcomes were self-reported; expectation effects on self-reported pain, function, and global change are well described. Because participants were unblinded and all outcomes were self-reported, expectation effects may have contributed to the observed differences, alongside secular changes and residual or unmeasured confounding. In addition, the ÖMPQ contains pain, function, and work-related items as well as psychosocial items, so its agreement with the NPRS and MSK-HQ does not constitute independent evidence against measurement bias. The Arabic ÖMPQ was validated in people with low back pain [36]; its measurement properties across other pain sites and over time were not examined in this study. Employment status was not recorded, so we cannot report how work-related items were answered by participants who were not in paid employment or examine whether this affected total scores. Safety was not systematically assessed: no structured adverse-event ascertainment procedure was used, the escalation criteria were not monitored by a structured procedure, and the reason for the one hospital admission is unknown. The study therefore cannot establish the safety of either pathway.

Fifth, the cohorts were not matched by calendar period: usual care ran from October 2025 to February 2026, and stratified care ran from February to August 2026. Recruitment was paused during Ramadan and Eid al-Fitr; the pause applied only to new recruitment, so 25 stratified-care participants recruited beforehand had intervention and follow-up periods that overlapped Ramadan, which is associated with changes in diet, chronotype, and healthcare-use timing [47,48]. The stratified-care follow-up also extended into the Saudi summer; higher temperatures and humidity are associated with lower physical activity in the Gulf region [49]. These unmatched calendar periods could have influenced engagement or self-reported outcomes in either direction; this non-randomized design cannot separate such temporal and seasonal effects from the pathway effect.

### 4.3. Findings That Converge with the Existing Literature

One finding aligned with evidence from the STarT MSK cluster-randomized trial by Hill et al. [17]. That trial reported higher patient satisfaction with stratified care, despite no significant differences in patient-reported clinical outcomes. Similarly, the present study found higher satisfaction in the stratified-care cohort. This consistency suggests that bundled, structured care may be associated with a better patient experience of care; however, the mechanism underlying the higher satisfaction cannot be determined from the present unblinded, non-randomized design.

### 4.4. Strengths

The study has several methodological strengths. Outcomes were self-completed using the same electronic assessment procedure at every time point in both cohorts, reducing the potential for differential measurement or assessor influence. This consistency is an advantage over some comparable before-and-after evaluations, including Gomes et al. [20], in which a research assistant collected usual-care data and treating physiotherapists collected intervention-cohort data. Nevertheless, identical outcome administration cannot eliminate reporting or expectation effects because participants knew what care they received. Retention was high (89.0% at 12 weeks) and did not differ significantly between cohorts. We used three instruments, and all three moved in the same direction. Because the ÖMPQ includes pain and function items and therefore overlaps with the NPRS and MSK-HQ, this agreement is not independent evidence against measurement artifact or expectation effects. The analysis used LMM with a justified random-effects structure, retained all available observations, and found that estimates were materially consistent across the sensitivity analyses. We are unaware of a previous published evaluation of a STarT MSK-informed, hybrid self-management pathway in Saudi primary care.

### 4.5. Limitations

Because the cohorts were recruited sequentially rather than through randomization, the pathway effect cannot be separated from secular changes, differences in case mix, and evolving clinical practice over the same period. Baseline differences were observed in age, pain intensity, prior physiotherapy, and the distribution of referral pain site. Although estimates remained in the same direction and statistically significant after adjustment, the adjustment addresses only measured covariates and cannot remove confounding inherent in a non-randomized design. All outcomes were self-reported by unblinded participants, and expectation effects cannot be excluded, as discussed above. Although the study recorded physician management decisions at the index consultation, it did not verify whether imaging or conventional physiotherapy referrals were completed and did not systematically capture subsequent medication use, routine-care consultation frequency, or treatments delivered outside the study pathway. Consequently, we could not comprehensively compare actual co-intervention exposure between cohorts. No usual-care participant crossed over to the stratified-care pathway or received the eSELF-MSK program, and we identified no such crossover during regular follow-up contact. However, because physician training occurred while some usual-care participants remained in outcome follow-up, we cannot completely exclude unrecorded reconsultation with a trained physician outside the study pathway.

Furthermore, because model-based baseline NPRS scores differed significantly between cohorts (*p* = 0.003), and baseline ÖMPQ (*p* = 0.156) and MSK-HQ (*p* = 0.181) scores differed numerically without reaching significance, we fitted post hoc, baseline-adjusted ANCOVA models as an additional sensitivity analysis at 12 weeks (Table 5). The adjusted between-cohort differences remained in the same direction and were statistically significant for all three outcomes, indicating that the observed 12-week differences were robust to adjustment for measured baseline outcome scores. However, this post hoc analysis does not eliminate regression to the mean, residual confounding, or confounding by unmeasured factors.

Eligibility was restricted to low-risk patients, so the findings do not extend to medium- or high-risk patients, who constituted approximately 79% of those screened. This was contrary to our expectation at the time of study design, based on the prior stratified-care literature, that low-risk patients would represent the largest share of primary-care MSK presentations. A similar pattern has been observed elsewhere: in a recent Australian feasibility trial of STarT MSK–guided telehealth stratified care, no intervention participant was classified as low risk [26]. As discussed above, this narrower population may also partly explain the larger effect observed here relative to trials enrolling all risk groups, a possibility we cannot distinguish from a true generalizability limitation with the present data.

Additionally, the study was conducted in a single center, which limits generalizability to other Saudi settings with different resources and referral pathways. Although consecutive patients were screened and 136 of 155 eligible low-risk patients were enrolled, participants were not compared with the wider clinic population, so their representativeness of patients with musculoskeletal pain in Saudi primary care is unknown. Participants who did not complete the six-week assessment were not recontacted at twelve weeks, so missingness was partly determined by the follow-up procedure; the missing-at-random assumption underlying the analysis therefore cannot be verified. This was a pragmatic decision by the research team, based on the assumption that participants who did not respond at 6 weeks would not respond at 12 weeks, rather than a prespecified protocol rule, and it may have produced avoidable missing data. Delta-adjusted multiple imputation and non-responder imputation for the responder analyses (Results 3.5) indicated that the conclusions were robust to substantial departures from missing-at-random, but these remain assumptions and cannot exclude bias. A descriptive comparison showed that non-completers were somewhat older, reported greater baseline pain, and were more often enrolled in the stratified-care cohort than completers (standardized differences exceeding 0.20). However, this comparison was underpowered given the 15 non-completers. The consistency of the complete-case analysis with the primary models provides some, though not conclusive, reassurance. A multiple imputation sensitivity analysis, pooling estimates across 30 imputed datasets using Rubin’s rules, produced results consistent with the primary mixed-effects model (Table 5), providing additional reassurance against bias from missing follow-up data. Some referral pain site subgroups had fewer than 10 participants, so the exploratory subgroup analysis is severely underpowered and serves only to generate hypotheses. Finally, follow-up was limited to 12 weeks, so this study cannot determine whether the observed differences persist beyond that point. Future studies need longer follow-up. No economic evaluation was undertaken; therefore, the cost-effectiveness and resource implications of implementing the pathway could not be determined.

## 5. Conclusions

Implementation of the bundled STarT MSK-guided eSELF-MSK pathway was associated with greater improvements in pain, MSK health, and psychosocial risk and with higher patient satisfaction than usual care among adults classified as low risk. These findings reflect the complete pathway and cannot isolate the contribution of risk classification, clinician training, physiotherapy assessment, education, exercise, or virtual follow-up. Because the cohorts were non-randomized, participants were unblinded, and outcomes were self-reported, the observed differences may also reflect expectation effects, secular changes, differences in usual care, and residual or unmeasured confounding. Further randomized or more rigorously controlled evaluation is required, and the present findings do not establish effectiveness or the optimal number of treatment contacts.

## Figures and Tables

**Table 3:** Responder analyses at 12 weeks.

Definition	Usual Care n/N (%)	Stratified n/N (%)	Risk Difference, % (95% CI)	Risk Ratio (95% CI)	Odds Ratio (95% CI)
NPRS reduction ≥ 2 points (post hoc)	15/58 (25.9)	43/63 (68.3)	+42.4 (+26.3 to +58.5)	2.64 (1.65–4.21)	6.16 (2.79–13.60)
MSK-HQ increase ≥ 6 points (post hoc)	20/58 (34.5)	39/63 (61.9)	+27.4 (+10.3 to +44.6)	1.80 (1.20–2.69)	3.09 (1.47–6.49)
MSK-HQ increase ≥ 6.83 points (post hoc)	19/58 (32.8)	38/63 (60.3)	+27.6 (+10.5 to +44.6)	1.84 (1.21–2.80)	3.12 (1.48–6.57)

**Table 4:** Satisfaction and global rating of change at 12 weeks.

Response	Usual Care (n = 58)	Stratified Care (n = 63)	Test
**Satisfaction with care (n = 121)**			
Very dissatisfied	3 (5.2)	2 (3.2)	
Dissatisfied	7 (12.1)	0 (0.0)	
Neither	18 (31.0)	5 (7.9)	
Satisfied	14 (24.1)	28 (44.4)	
Very satisfied	16 (27.6)	28 (44.4)	
Median (IQR)	4 (3–5)	4 (4–5)	U = 1176.0; *p* < 0.001
**GROC (n = 121)**			
−5 to −1 (worse)	10 (17.2)	3 (4.8)	
0 (no change)	12 (20.7)	3 (4.8)	
+1 to +2 (minimally better)	11 (19.0)	11 (17.5)	
+3 to +5 (clearly better)	25 (43.1)	46 (73.0)	
Median (IQR)	2 (0–4)	4 (2–5)	U = 1188.5; *p* < 0.001

**Table 5:** Sensitivity analyses of between-cohort estimate at 12 weeks.

Model or Analysis Set	NPRS (95% CI)	MSK-HQ (95% CI)	ÖMPQ (95% CI)
Primary model (random intercept and slope, unadjusted)	−2.12 (−2.95 to −1.29)	+7.08 (+3.70 to +10.47)	−27.83 (−37.90 to −17.76)
**Single-covariate adjustments (cohort-by-time contrast)**			
Adjusted for referral pain site	−2.12 (−2.94 to −1.30)	+7.11 (+3.75 to +10.46)	−27.86 (−37.84 to −17.88)
Adjusted for age	−2.12 (−2.94 to −1.29)	+7.08 (+3.73 to +10.44)	−27.88 (−37.86 to −17.90)
Adjusted for previous physiotherapy	−2.12 (−2.94 to −1.30)	+7.08 (+3.73 to +10.44)	−27.83 (−37.80 to −17.85)
**Jointly adjusted models (cohort-by-time contrast)**			
Model A: jointly adjusted for age, gender, referral pain site, previous physiotherapy, and symptom duration (main effects)	−2.14 (−2.97 to −1.30)	+7.11 (+3.73 to +10.50)	−28.10 (−38.19 to −18.01)
Model B: Model A plus each covariate × time interaction	−2.09 (−3.01 to −1.17)	+7.64 (+3.82 to +11.47)	−27.12 (−38.67 to −15.57)
**Alternative model specification and data handling (cohort-by-time contrast)**			
Random intercept only, no random slope	−2.11 (−2.86 to −1.36)	+7.06 (+4.03 to +10.08)	−27.99 (−36.75 to −19.22)
Excluding standardized residuals beyond 3 SD	−2.21 (−3.02 to −1.40)	+7.49 (+4.30 to +10.68)	−27.87 (−37.84 to −17.90)
Complete cases only (363 observations, 121 participants)	−2.18 (−3.00 to −1.35)	+7.19 (+3.78 to +10.59)	−27.90 (−38.05 to −17.75)
**Baseline-adjusted 12-week mean difference (different estimand)**			
ANCOVA (baseline outcome score entered as covariate rather than as a modeled time point), 12-week adjusted mean difference	−1.67 (−2.48 to −0.87)	+6.11 (+2.93 to +9.28)	−25.98 (−35.82 to −16.14)
Model C: baseline outcome score and baseline × time as covariates (6- and 12-week observations only), 12-week adjusted mean difference	−1.65 (−2.43 to −0.86)	+6.16 (+3.22 to +9.09)	−25.76 (−34.52 to −17.00)
Model D: baseline outcome score, age, gender, referral pain site, previous physiotherapy, and symptom duration, each with its interaction with time (6- and 12-week observations only), 12-week adjusted mean difference	−1.60 (−2.46 to −0.74)	+6.59 (+3.31 to +9.87)	−25.57 (−35.37 to −15.77)
**Missing-data analysis (cohort-by-time contrast)**			
Multiple imputation (30 imputed datasets, Rubin’s rules pooling)	−2.01 (−2.83 to −1.20)	+6.80 (+3.37 to +10.23)	−27.39 (−37.32 to −17.47)

**Table 6:** Selected comparative evidence on stratified care and digital self-management.

Study	Design	N	Intervention Tested	Reported Clinical Effect (95% CI)
Hill et al. 2022 (STarT MSK) [17]	Cluster RCT, UK	1211	Keele STarT MSK Tool with GP decision-support template	Time-averaged pain difference: −0.16 (−0.65 to 0.34)
Hill et al. 2011 (STarT Back) [42]	RCT, UK	851	STarT Back Tool with matched physiotherapy	RMDQ improvement difference: +1.81 (1.06 to 2.57) at 4 months and +1.06 (0.25 to 1.86) at 12 months
Foster et al. 2014 (IMPaCT Back) [19]	Sequential cohort, UK	922	STarT Back implementation in family practice	Difference in pain improvement: +0.29 (−0.05 to 0.63)
Gomes et al. 2024 (SPLIT) [20]	Sequential cohort, Portugal	447	Stratified care with a matched physiotherapy program	Adjusted pain difference: −0.88 (−1.18 to −0.57)
Van Tilburg et al. 2026 (SBPA) [41]	Cluster RCT, The Netherlands	139	Keele STarT MSK Tool with matched-treatment, blended-care physiotherapy	9-month adjusted mean difference: −0.91 (−7.39 to 5.56)
Valentijn et al. 2022 [43]	Meta-analysis of 56 RCTs	9359	Digital self-management for MSK pain	Pain SMD 0.19 (0.06 to 0.31)
Present study	Sequential cohort, Saudi Arabia	136	Bundled STarT MSK-guided low-risk pathway incorporating clinician training, physiotherapy assessment, education, exercise, and virtual follow-up	6-week differential change in NPRS: −2.34 (−3.05 to −1.64)

MSK = musculoskeletal; NPRS = Numeric Pain Rating Scale; RCT = randomized controlled trial; RMDQ = Roland–Morris Disability Questionnaire; SMD = standardized mean difference. The reported estimates are not directly comparable because the studies differed in design, population, comparator, outcome measure, follow-up period, and analytical method. Negative values favor the intervention when effects are expressed as differences in pain scores, whereas positive values favor the intervention when expressed as greater improvement or as a standardized effect. The present study estimate represents the cohort-by-time interaction at 6 weeks and should not be interpreted as the within-group improvement in the stratified-care cohort. This table excludes predictive-validity cohort studies of the Keele STarT MSK Tool (e.g., Vries et al.) [44]) and Beneciuk et al. [16], who validated the tool’s construct and predictive validity in a United States outpatient physical therapy cohort, because they report prognostic discrimination rather than the effect of stratified care on outcomes.

## Data Availability

The data presented in this study are not publicly available due to institutional restrictions on patient data sharing at Prince Sultan Military Medical City and applicable Saudi data protection regulations. Study data were collected and managed using REDCap electronic data capture tools, anonymized, stored on encrypted systems, and retained for five years in accordance with institutional policy.

## Supplementary Materials

The following supporting information can be downloaded at https://www.mdpi.com/article/10.3390/jcm15208105/s1. Supplementary Material S1, comprising the statistical analysis specification and reporting record, sensitivity, subgroup, and per-protocol analyses, and Figure S1 and Tables S1–S4 are provided with this submission. The references cited in Supplementary Material S1 are [13,31,34,35,39]. The completed STROBE checklist for cohort studies is submitted separately with this manuscript for editorial and peer-review reference. Other study materials described in this manuscript, including the eSELF-MSK program protocol and physician training materials, are available from the corresponding author upon reasonable request.

## Abbreviations

ANCOVA = Analysis of Covariance; BMI = Body Mass Index; CI = Confidence Interval; GROC = Global Rating of Change; IQR = Interquartile Range; LMM = Linear Mixed-Effects Models; MSK = Musculoskeletal; MSK-HQ = Musculoskeletal Health Questionnaire; NPRS = Numeric Pain Rating Scale; ÖMPQ = Örebro Musculoskeletal Pain Questionnaire; PSMMC = Prince Sultan Military Medical City; RCT = Randomized Controlled Trial; RMDQ = Roland–Morris Disability Questionnaire; SMD = Standardized Mean Difference; STROBE = Strengthening the Reporting of Observational Studies in Epidemiology.
