# Supplementary material for: Stratified Care for Musculoskeletal Conditions in Primary Care: A Prospective Sequential Cohort Study of the STarT MSK Tool and the eSELF-MSK Virtual Self-Management Program
Source: J Clin Med. 2026 Oct 19;15(20):8105. doi: 10.3390/jcm15208105 (PMC13706539; doi:10.3390/jcm15208105)
Supplement: Supplementary file 1 [file jcm-15-08105-s001.zip › jcm-4578105-supplementary.pdf]

Supplementary Material S1: Statistical analysis specification and reporting record

Supplementary Material for manuscript jcm-4578105

Stratified Care for Musculoskeletal Conditions in Primary Care: A Prospective Sequential Cohort Study of the STarT MSK Tool and the eSELF-MSK Virtual Self-Management Program

Purpose and Study Design

This document provides a reproducible record of the statistical analysis of a non-randomized sequential cohort study comparing usual care (Cohort 1) with a stratified care pathway incorporating the eSELF-MSK virtual self-management program (Cohort 2). Its purpose is to state precisely what was analyzed, how, and on what basis each analytic decision was made, so that the reported results can be traced from the raw data to the final estimates. It records the prespecified analysis, the conducted analysis, and the classification of every reported result.

Classification used in this report

Each analysis reported in the manuscript is assigned one of the following classifications. The applicable classification is stated in the relevant section of this record.

Classification	Definition
Prespecified	Specified in the study proposal approved by the Institutional Review Board before data collection.
Final implementation	Operational detail required to fit a prespecified analysis, decided during analysis on methodological grounds.
Deviation	Departure from the prespecification, with the reason recorded.
Post hoc	Analysis chosen after the data had been examined.
Sensitivity	Alternative model or analysis set used to test the robustness of a primary result.
Exploratory	Analysis added during study conduct before examination of the relevant outcome results; interpreted as hypothesis-generating.

Outcomes and their analytic role

Outcome	Scale and direction	Classification	Analysis applied
NPRS	0–10; lower is better	Prespecified	Linear mixed-effects model; responder analysis at ≥2-point reduction(the ≥2-point responder threshold is not in the proposal and is post hoc)
MSK-HQ	0–56; higher is better	Prespecified	Linear mixed-effects model; responder analyses post hoc
ÖMPQ	0–210; lower is better	Prespecified (secondary outcome in the proposal)	Linear mixed-effects model only
Patient Satisfaction	5-point ordinal, 12 weeks	Prespecified secondary	Category n (%), median (IQR), Mann–Whitney U test
GROC	−5 to +5, 12 weeks	Post hoc	Category n (%), median (IQR), Mann–Whitney U test; binary threshold post hoc

GROC = Global Rating of Change; MSK-HQ = Musculoskeletal Health Questionnaire; NPRS = Numeric Pain Rating Scale; ÖMPQ = Örebro Musculoskeletal Pain Questionnaire. The MSK-HQ was developed and validated by Hill et al. [34]; responsiveness and the minimal important change estimate are reported by Price et al. [39]; the Arabic version and its measurement properties are reported by Algarni et al. [35].

Analysis sets and observations

Participants contributed all available observations. Participants were not excluded from the longitudinal models solely because a follow-up assessment was missing. Denominators are stated for every analysis.

Analysis set	Usual care	Stratified care	Total
Enrolled	63	73	136
Observed at baseline	63	73	136
Observed at 6 weeks	61	68	129
Observed at 12 weeks	58	63	121
Complete data at all three timepoints	58	63	121

Of 750 patients screened across the two cohorts, 155 (20.7%) were classified as low risk on the Keele STarT MSK tool and were therefore eligible. Nineteen eligible patients were not enrolled because they did not attend the clinic after triage or declined to participate. All 136 enrolled participants had a STarT MSK total score of 4 or below (observed range 1 to 4), consistent with the eligibility criterion.

Software and timing

Analyses were conducted in IBM SPSS Statistics version 31. Follow-up assessments were conducted at 6 and 12 weeks rather than at 1 and 3 months as originally proposed. The supervisory team agreed to this change before recruitment commenced, so the first assessment would coincide with discharge from the eSELF-MSK program at 6 weeks and the second would capture outcomes six weeks after discharge. This is recorded as a deviation agreed prior to data collection.

The delta-adjusted multiple-imputation and non-responder-imputation sensitivity analyses were conducted using Python 3.11 with statsmodels version 0.15.0. The mixed-effects model specification was aligned with the primary model implemented in SPSS.

All outcome measures were self-completed by participants using an identical electronic form at every timepoint in both cohorts. No assessor administered any outcome measure, and the mode of administration did not differ between cohorts.

Descriptive and baseline analyses

The distribution of each continuous baseline variable was assessed within each cohort using the Shapiro–Wilk test, histograms, normal Q–Q plots, and measures of skewness and kurtosis. MSK-HQ and ÖMPQ satisfied the normality assumption in both cohorts and were compared using the independent-samples t-test with Cohen's d. Levene's test was non-significant for both, and the equal-variances assumption was met. Age, body mass index, NPRS, and STarT MSK total deviated from normality in at least one cohort and were compared using the Mann–Whitney U test; results are summarized as median (interquartile range).

Categorical variables were compared using the Pearson chi-square test with Cramér's V. Fisher's exact test was used when any expected cell count was below 5, as indicated in the table. Continuity-corrected chi-square values are not reported. No adjustment for multiple comparisons was applied to the baseline table, which is descriptive; effect sizes are reported alongside p-values because, with this sample size, differences of limited clinical relevance can attain statistical significance.

A sequential cohort design does not produce equivalent groups, and non-significant tests do not demonstrate equivalence. Baseline differences are therefore described and addressed through sensitivity analysis rather than characterized as a comparability issue.

Longitudinal models as conducted

Model form

A separate linear mixed-effects model was fitted for NPRS, MSK-HQ, and ÖMPQ, with estimates obtained by restricted maximum likelihood. Fixed effects were cohort, time entered as a categorical factor with levels baseline, 6 weeks, and 12 weeks, and the cohort-by-time interaction. Participant was the clustering unit. No covariates were included in the primary models, consistent with the IRB-approved study proposal. A numeric copy of the time variable was used solely to define the participant-specific random slope; time remained categorical in the fixed effects throughout.

Random effects structure

The IRB-approved study proposal allowed participant-specific random slopes to be added where appropriate. A random-intercept model was compared with one that additionally included a random linear slope for time, with an unstructured covariance matrix for the random effects, using a likelihood-ratio test with 2 degrees of freedom.

Outcome	−2 RLL, intercept only	−2 RLL, with slope	Likelihood ratio	P
NPRS	1584.52	1558.29	26.23	<.001
MSK-HQ	2674.53	2663.16	11.37	.003
ÖMPQ	3532.81	3516.97	15.85	<.001

−2 RLL = −2 restricted log-likelihood. All three tests exceed the critical value of 5.99 on 2 degrees of freedom. The models reported throughout therefore include a participant-specific random intercept and random linear slope for time. This reflects the final implementation of a prespecified provision.

Estimands and interpretation

The omnibus cohort-by-time interaction was the principal test of differential trajectories for each outcome. The estimands of interest were the between-cohort differences in change from baseline at 6 and 12 weeks. Twelve-week contrasts are reported directly from the fixed-effects table. Six-week contrasts were obtained as custom linear contrasts of the fixed-effect parameters, providing standard errors and confidence intervals estimated from the model covariance matrix (NPRS $t(222.7) = -6.55$; MSK-HQ $t(226.9) = 5.11$; ÖMPQ $t(235.3) = -5.87$; all $p < .001$). Signs are interpreted with respect to each scale's direction: for NPRS and ÖMPQ, a negative contrast favors stratified care, whereas for MSK-HQ, a positive contrast favors stratified care.

Assumption checks and diagnostics

Diagnostics were conducted separately for each outcome using uniquely named residual and predicted-value variables, so that each diagnostic can be attributed to its corresponding model without ambiguity.

Outcome	Residual mean	SD	Skewness	Kurtosis	|z| > 3
NPRS	0.000	1.125	+0.27	+0.58	3 (0.8%)
MSK-HQ	0.000	4.176	−0.54	+0.76	2 (0.5%)
ÖMPQ	0.000	10.988	+0.22	+0.28	1 (0.3%)

Residual distributions were examined using histograms, normal Q–Q plots, skewness and kurtosis. All skewness and kurtosis values fell within ±1. Homoscedasticity was assessed using plots of residuals against predicted values for each outcome; no marked funneling or systematic pattern was observed.

The influence of outlying observations was assessed by identifying standardized residuals exceeding three standard deviations and refitting each model after excluding those observations. SPSS MIXED did not provide case-level influence diagnostics suitable for these models; therefore, stability of the cohort-by-time estimates after exclusion was used as the influence assessment. Between one and three of the 386 observations met the exclusion criterion for each outcome, and the refitted estimates are presented in Table 5. This is recorded as a final implementation decision.

Missing data

Participants who did not complete the 6-week assessment were not recontacted at 12 weeks. All seven participants missing at 6 weeks were consequently also missing at 12 weeks, with a further eight losses occurring between 6 and 12 weeks. Missingness was therefore monotone and partly determined by the follow-up procedure.

Follow-up procedure. Participants who had not completed an assessment were sent three reminder messages at different times, followed by a telephone call, a one-day wait, and a final message. Participants who did not complete the 6-week assessment after this sequence were not approached again for the 12-week assessment; this was a pragmatic decision by the investigator and not a prespecified protocol rule, and it may have produced avoidable missing data. Of the 15 participants without a 12-week outcome (usual care 5, stratified care 10), 7 were also missing at 6 weeks and 8 had completed the 6-week assessment; reasons are given in the Results (Section 3.1) of the manuscript.

The primary mixed models use all available repeated outcome observations and provide likelihood-based estimates under a missing-at-random assumption conditional on the variables represented in the model. No single imputation or ad hoc replacement was used.

The IRB-approved study proposal anticipated multiple imputation for missing longitudinal data. An initial attempt using SPSS Multiple Imputation (20 imputed datasets, monotone method, wide format) did not produce valid mixed-model estimates pooled across imputations, and those results were not reported. Multiple imputation was subsequently repeated using chained-equations regression imputation in SPSS (30 imputed datasets), with missing 6- and 12-week values for NPRS, MSK-HQ, and ÖMPQ imputed from baseline outcome scores, cohort, age, gender, nationality, symptom duration, body mass index, referral pain site, and prior physiotherapy. The primary mixed-effects model (fixed effects for cohort, categorical time, and their interaction, with a participant-specific random intercept and slope) was refitted to each imputed dataset, and cohort-by-time estimates at 12 weeks were pooled across imputations using Rubin's rules. Pooled estimates were closely consistent with the primary likelihood-based mixed-effects model (NPRS, −2.01; 95% CI, −2.83 to −1.20; MSK-HQ, +6.80; 95% CI, +3.37 to +10.23; ÖMPQ, −27.39; 95% CI, −37.32 to −17.47; all $p < 0.001$) and are reported in Table 5. A separate complete-case analysis of 121 participants with observations at all three time points was conducted as an additional missing-data sensitivity analysis.

Delta-adjusted (tipping-point) analysis. To examine departures from the missing-at-random assumption (sensitivity analysis, post hoc, added in response to peer review), the multiple-imputation analysis was repeated with a fixed shift (delta) added to the imputed 6- and 12-week values in the stratified-care cohort only, in the direction of a worse outcome (higher for NPRS and ÖMPQ, lower for MSK-HQ). Missing values were imputed by sequential Bayesian regression on baseline outcome score, cohort, age, gender, referral pain site, previous physiotherapy, and symptom duration (and on the 6-week value for the 12-week imputation);

30 imputed datasets were analysed with the primary mixed-effects model and pooled with Rubin's rules. The tipping point is the smallest shift at which the 95% confidence interval includes zero. Results are in Table S1 (part A). For scale, the shifts at which significance was lost (6 NPRS points, 20 MSK-HQ points, and 80 ÖMPQ points) correspond to approximately 3.4, 2.3, and 2.9 baseline standard deviations, respectively.

Non-responder imputation. In the responder analyses, participants with missing 12-week data were counted as non-responders, which gives the conservative case in which every missing participant failed to respond (Table S1, part B). The complete-case responder results are those in Table 3 of the manuscript.

Table S1 (part A). Delta-adjusted multiple imputation: 12-week cohort-by-time contrast (stratified minus usual care), 95% CI.

Outcome	δ = 0 (missing at random)	Largest shift retaining significance	First shift losing significance
ÖMPQ (higher imputed = worse)	−28.22 (−38.48 to −17.96)	δ = +60: −16.86 (−29.41 to −4.31)	δ = +80: −12.80 (−26.96 to +1.36); p = 0.076
MSK-HQ (lower imputed = worse)	+7.20 (+3.83 to +10.56)	δ = −16: +4.29 (+0.47 to +8.10)	δ = −20: +3.57 (−0.55 to +7.69); p = 0.089
NPRS (higher imputed = worse)	−2.14 (−2.97 to −1.31)	δ = +5: −1.23 (−2.26 to −0.21)	δ = +6: −1.05 (−2.14 to +0.03); p = 0.058

Table S1 (part B). Responder analyses with missing 12-week data counted as non-response (n = 63 usual care; n = 73 stratified care).

Responder definition	Usual care n/N (%)	Stratified care n/N (%)	Risk difference, percentage points (95% CI)
NPRS reduction ≥ 2 points	15/63 (23.8)	43/73 (58.9)	+35.1 (+19.7 to +50.5)
MSK-HQ increase ≥ 6 points	20/63 (31.7)	39/73 (53.4)	+21.7 (+5.5 to +37.9)
MSK-HQ increase ≥ 6.83 points	19/63 (30.2)	38/73 (52.1)	+21.9 (+5.8 to +38.0)

Sample Size Rationale

An a priori calculation was conducted in G*Power version 3.1.9.4 using a repeated-measures within-between interaction as a planning approximation for the mixed-effects analysis. The calculation assumed a small effect (f = 0.15) based on evidence from the literature, particularly Foster et al. [31] and Hill et al. [13]; two-sided α = 0.05; 80% power; two cohorts; three time points; a correlation of 0.50 among repeated measurements; and a nonsphericity correction of 1.00. The required total sample was 74 participants (37 per cohort). Allowing for 20% attrition produced an overall recruitment target of approximately 93 participants. The study enrolled 136 participants (63 in usual care and 73 in stratified care), exceeding the overall recruitment target. This calculation was a planning approximation and did not guarantee adequate power for each of the three longitudinal outcomes separately.

Responder analyses

Responder analyses supplement rather than replace continuous-outcome models and are restricted to participants with sufficient observations to calculate change. NPRS response was defined as a reduction of at least 2 points; the ≥2-point threshold is not in the IRB-approved proposal and was selected post hoc. For MSK-HQ, two thresholds are reported transparently, and both are post hoc: an increase of at least 6 points, operationalizing the 5.5-point minimal important change estimate reported by Price et al. [39] in their evaluation of MSK-HQ responsiveness, and an increase of at least 6.83 points, corresponding to the Minimal Detectable Change (MDC) at the 95% confidence level reported for the Arabic version of the instrument by Algarni et al. [35]. The latter reflects measurement reliability and represents change beyond measurement error; it is not a minimal clinically important difference, and it shows that the conclusion does not depend on which threshold is applied. The two thresholds are close in magnitude, and reporting both demonstrates that the conclusion is unchanged whether response is defined by clinical importance or by change exceeding Arabic-specific measurement error.

Proportions were compared using the Pearson chi-square test, with Fisher's exact test available where cells were sparse; no expected cell count fell below five in any responder analysis. Risk differences, risk ratios and odds ratios are reported with 95% confidence intervals.

Satisfaction and global rating of change

Satisfaction was a prespecified ordinal secondary outcome measured at 12 weeks and was collected in both cohorts. It was summarized using response-category frequencies and the median and interquartile range, and cohorts were compared using the Mann–Whitney U test. The IRB-approved study proposal specified an independent-samples t test or a nonparametric equivalent when assumptions were not met; the nonparametric test was appropriate for a single ordinal item. Means and standard deviations were not used as the primary summaries.

GROC was not prespecified. Because no dated record shows that its analysis was planned before outcome data were examined, we classified it as post hoc and interpreted it exploratorily. The ≥+3 threshold was also post hoc.

Sensitivity analyses

Results are presented in Table 5. The primary model and the first six sensitivity analyses estimated the 12-week cohort-by-time contrast, representing the between-cohort difference in change from baseline. The post hoc ANCOVA estimated the baseline-adjusted between-cohort mean difference at 12 weeks and therefore represented a different estimand. The multiple-imputation analysis estimated the pooled 12-week cohort-by-time contrast across 30 imputed datasets using Rubin's rules. Robustness was evaluated using the magnitude,

direction, confidence interval, and clinical interpretation of the estimates rather than statistical significance alone. Covariate adjustment addresses only measured characteristics and cannot eliminate residual or unmeasured confounding inherent in the non-randomized design.

Jointly adjusted models (Models A–D). Four models were added in response to peer review (sensitivity analyses, post hoc) to address baseline imbalance between the cohorts. Model A added age (centered), gender, referral pain site, previous physiotherapy, and symptom duration (centered) as main effects to the primary model; Model B additionally included each of these covariates by time. Models A and B estimate the same cohort-by-time contrast as the primary model. Model C used the 6- and 12-week observations only, with the baseline outcome score (centered) and baseline by time as covariates and a participant random intercept; Model D additionally included the joint covariates, each with its interaction with time. Models C and D estimate baseline-adjusted 12-week mean differences, which is a different estimand. Covariates were selected on clinical grounds as plausible determinants of the outcomes, and their number was limited by the sample size. Results are in Table S2.

Table S2. Primary and jointly adjusted models: between-cohort estimates at 12 weeks (stratified minus usual care), 95% CI.

Model and estimand	ÖMPQ (0–210)	MSK-HQ (0–56)	NPRS (0–10)
Primary model: differential change	−27.83 (−37.90 to −17.76)	+7.08 (+3.70 to +10.47)	−2.12 (−2.95 to −1.29)
Model A: differential change	−28.10 (−38.19 to −18.01)	+7.11 (+3.73 to +10.50)	−2.14 (−2.97 to −1.30)
Model A: contemporaneous 12-week difference	−23.28 (−35.28 to −11.27); p < 0.001	+5.21 (+1.48 to +8.94); p = 0.007	−1.25 (−2.15 to −0.34); p = 0.007
Model B: differential change	−27.12 (−38.67 to −15.57)	+7.64 (+3.82 to +11.47)	−2.09 (−3.01 to −1.17)
Model B: contemporaneous 12-week difference	−22.00 (−34.76 to −9.23); p < 0.001	+5.48 (+1.53 to +9.42); p = 0.007	−1.18 (−2.14 to −0.22); p = 0.017
Model C: baseline-adjusted 12-week difference	−25.76 (−34.52 to −17.00)	+6.16 (+3.22 to +9.09)	−1.65 (−2.43 to −0.86)
Model D: baseline-adjusted 12-week difference	−25.57 (−35.37 to −15.77)	+6.59 (+3.31 to +9.87)	−1.60 (−2.46 to −0.74)

Differential change = cohort-by-time contrast (difference in change from baseline). All differential-change and baseline-adjusted estimates had $p < 0.001$. Negative values favor stratified care for ÖMPQ and NPRS; positive values favor stratified care for MSK-HQ.

Exploratory subgroup and per-protocol analyses. In response to peer review, the primary model (unadjusted; random intercept and slope) was fitted separately by sex and by referral pain site (back, knee, and shoulder; neck, n = 10, and multisite, n = 9, were too small to analyze), and the cohort-by-time-by-subgroup interaction was tested (Type III F test). A per-protocol analysis excluded the 5 stratified-care participants who did not complete the program, defined as attendance at the initial in-person assessment and at least three of the five scheduled virtual follow-up sessions (68 of 73 completed). These analyses are post hoc, underpowered, and hypothesis-generating; excluding non-completers may introduce selection bias, so the per-protocol analysis

is supportive rather than causal. The NPRS models by pain site used a random intercept only because models with a random slope did not converge for knee and back pain. Results are in Table S3.

Table S3. Exploratory subgroup and per-protocol analyses: difference in change from baseline to 12 weeks (stratified minus usual care), 95% CI.

Analysis	n usual / stratified	ÖMPQ	MSK-HQ	NPRS
Overall (primary model)	63 / 73	−27.83 (−37.90 to −17.76); p < 0.001	+7.08 (+3.70 to +10.47); p < 0.001	−2.12 (−2.95 to −1.29); p < 0.001
Men	32 / 27	−24.10 (−41.20 to −7.01); p = 0.007	+9.13 (+3.58 to +14.69); p = 0.002	−2.06 (−3.46 to −0.66); p = 0.005
Women	31 / 46	−30.19 (−42.84 to −17.55); p < 0.001	+5.23 (+0.91 to +9.56); p = 0.018	−2.23 (−3.28 to −1.18); p < 0.001
Knee	12 / 29	−26.27 (−42.71 to −9.83); p = 0.003	+7.58 (+1.31 to +13.86); p = 0.019	−1.17 (−2.42 to +0.08); p = 0.066 †
Shoulder	7 / 15	−55.10 (−87.72 to −22.48); p = 0.002	+13.00 (+2.23 to +23.77); p = 0.020	−2.70 (−5.28 to −0.12); p = 0.041 †
Back	29 / 25	−17.93 (−35.34 to −0.51); p = 0.044	+3.64 (−1.81 to +9.09); p = 0.186	−1.54 (−2.75 to −0.32); p = 0.014 †
Per-protocol (excludes 5 non-completers)	63 / 68	−27.75 (−37.84 to −17.66); p < 0.001	+7.10 (+3.71 to +10.49); p < 0.001	−2.14 (−2.98 to −1.31); p < 0.001
Interaction: cohort × time × sex (2 df)		p = 0.607	p = 0.545	p = 0.528
Interaction: cohort × time × pain site (4 df)		p = 0.261	p = 0.089	p = 0.344

† Random-intercept model (variance components). All analyses are exploratory and underpowered. Sex as recorded (59 men, 77 women).

Departures from the IRB-approved proposal. The proposal submitted in April 2025 was compared with the study as conducted (Table S4). The Global Rating of Change and the responder, delta-adjusted, non-responder, jointly adjusted, subgroup, and per-protocol analyses were not in the proposal; the last five were added in response to peer review.

Table S4. Comparison of the IRB-approved proposal (April 2025) with the study as conducted.

Item	Proposal (April 2025)	Study as conducted
Comparison group	Proposal abstract: outcomes compared with a matched historical usual-care cohort	Usual-care cohort enrolled prospectively and sequentially before the stratified-care phase
Recruitment period	June to August 2025	October to November 2025 (usual care); February–April 2026. (stratified care)
Follow-up time points	Baseline, 1 month, and 3 months	Baseline, 6 weeks, and 12 weeks
Primary outcomes	Pain intensity (NRS) and MSK-HQ	NPRS and MSK-HQ
Secondary outcomes	ÖMPQ (described in the outcome-measures section and named in the statistical analysis section as psychosocial risk, analyzed with the same mixed-effects models); patient satisfaction at 3 months.	ÖMPQ analyzed with the same mixed-effects models and reported alongside the NPRS and MSK-HQ; satisfaction at 12 weeks; Global Rating of Change added
Sample size	G*Power version 3.1.9.4, repeated-measures within-between ANOVA approximation, f = 0.15, alpha 0.05, power 80%: 90 participants, 114 after 20% dropout (the G*Power input in Appendix 2 used two measurements)	Recalculated for three measurements (correlation 0.5 among repeated measures): 74, 93 after dropout; 136 enrolled
Main analysis	Linear mixed model with treatment, time, and treatment-by-time; random intercepts and, where appropriate, slopes; multiple imputation under MAR; sensitivity analyses “may be conducted”	As proposed (REML; random intercept and slope)
Responder analyses	Not specified	Added; NPRS and MSK-HQ thresholds from the literature; all post hoc
Global Rating of Change	Not specified	Added; exploratory
Analyses added at peer review	Not specified	Models A–D, delta-adjusted multiple imputation, non-responder imputation, subgroup and per-protocol analyses (post hoc)

Inference and reporting conventions

- All tests were two-sided with $\alpha = 0.05$ and 95% confidence intervals.
- Chi-square results reported from the Pearson row; Fisher's exact test reported and identified where expected counts fell below five.
- Effect sizes reported alongside p-values for all baseline comparisons.
- No multiplicity adjustment was applied across the three longitudinal outcomes or the two postbaseline contrasts. Findings were therefore interpreted using effect estimates, 95% confidence intervals, clinical magnitude, and consistency across outcomes rather than p-values alone.
- Post-hoc and exploratory analyses were identified as such wherever reported and interpreted as hypothesis-generating.
- Estimates reported to two decimal places; p-values below .001 reported as < .001.

References

13. Hill, J.; Garvin, S.; Chen, Y.; Cooper, V.; Wathall, S.; Saunders, B.; Lewis, M.; Protheroe, J.; Chudyk, A.; Dunn, K. Stratified primary care versus non-stratified care for musculoskeletal pain: findings from the STarT MSK feasibility and pilot cluster randomized controlled trial. BMC Fam. Pract. **2020**, 21, 1-18. https://doi.org/10.1186/s12875-019-1074-9.

31. Foster, N.E.; Dunn, K.M.; Protheroe, J.; Hill, J.C.; Lewis, M.; Saunders, B.; Jowett, S.; Hennings, S.; Campbell, P.; Bromley, K. Stratified primary care for adults with musculoskeletal pain: the STarT MSK research programme including RCTs. *Program. Grants Appl. Res.* **2023**, 11, 1-103. https://doi.org/10.3310/fbvx4177.

34. Hill, J.C.; Kang, S.; Benedetto, E.; Myers, H.; Blackburn, S.; Smith, S.; Dunn, K.M.; Hay, E.; Rees, J.; Beard, D. Development and initial cohort validation of the Arthritis Research UK Musculoskeletal Health Questionnaire (MSK-HQ) for use across musculoskeletal care pathways. BMJ Open **2016**, 6, e012331.

35 Algarni, F.S.; Alotaibi, A.N.; Altowaijri, A.M.; Al-Sobayel, H. Cross-cultural adaptation and validation of the Arabic version of musculoskeletal health questionnaire (MSK-HQ-Ar). Int. J. Environ. Res. Public Health **2020**, 17, 5168.

39 Price, A.J.; Ogollah, R.; Kang, S.; Hay, E.; Barker, K.L.; Benedetto, E.; Smith, S.; Smith, J.; Galloway, J.B.; Ellis, B. Determining responsiveness and meaningful changes for the Musculoskeletal Health Questionnaire (MSK-HQ) for use across musculoskeletal care pathways. BMJ Open **2019**, 9, e025357.

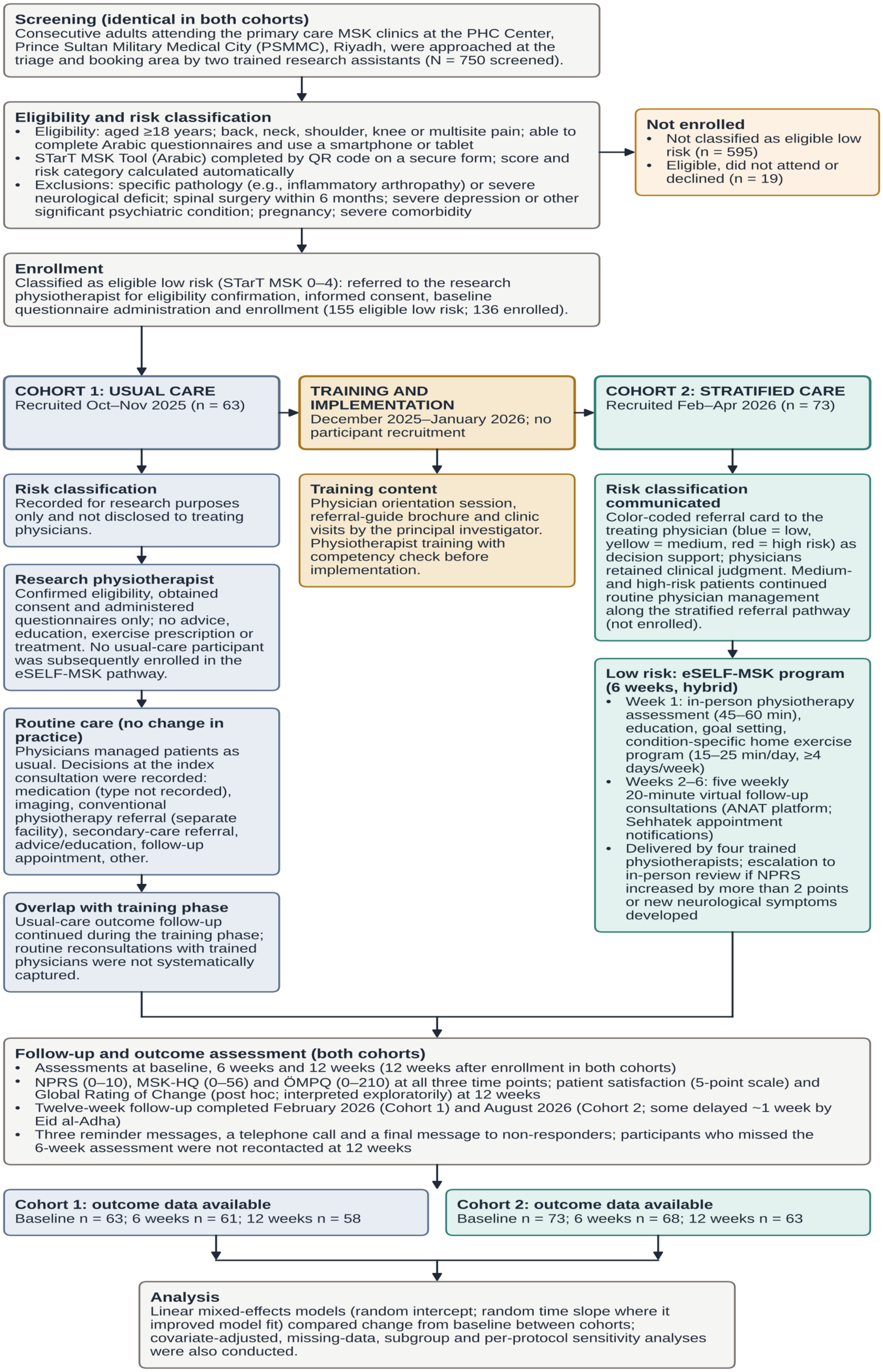

Figure S1. Detailed study workflow, care pathways, follow-up, and analysis. MSK, musculoskeletal; NPRS, Numerical Pain Rating Scale; MSK-HQ, Musculoskeletal Health Questionnaire; ÖMPQ, Örebro Musculoskeletal Pain Questionnaire; STarT MSK, Keele STarT Musculoskeletal Tool.
